# Decreased cerebrospinal fluid kynurenic acid in epileptic spasms: A biomarker of response to corticosteroids

**DOI:** 10.1016/j.ebiom.2022.104280

**Published:** 2022-09-26

**Authors:** Jingya Yan, Kavitha Kothur, Emily A. Innes, Velda X. Han, Hannah F. Jones, Shrujna Patel, Erica Tsang, Richard Webster, Sachin Gupta, Christopher Troedson, Manoj P. Menezes, Jayne Antony, Simone Ardern-Holmes, Esther Tantsis, Shekeeb Mohammad, Louise Wienholt, Ananda S. Pires, Benjamin Heng, Gilles J. Guillemin, Anna Guller, Deepak Gill, Sushil Bandodkar, Russell C. Dale

**Affiliations:** a Kids Neuroscience Centre, The Children's Hospital at Westmead, Faculty of Medicine and Health, University of Sydney, NSW, Australia; b Department of Biochemistry, The Children's Hospital at Westmead, NSW, Australia; c TY Nelson Department of Neurology and Neurosurgery, The Children's Hospital at Westmead, The University of Sydney, Westmead, New South Wales, Australia; d Khoo Teck Puat-National University Children's Medical Institute, National University Health System, Singapore, Singapore; e Starship Hospital, Centre for Brain Research, Faculty of Medical and Health Sciences, University of Auckland, New Zealand; f Clinical School, The Children's Hospital at Westmead, Faculty of Medicine and Health, University of Sydney, NSW, Australia; g Department of Clinical Immunology and Allergy, Royal Prince Alfred Hospital, Camperdown, NSW, Australia; h Neuroinflammation Group, Macquarie Medical School, Faculty of Medicine, Health and Human Sciences, Macquarie University, NSW, Australia; i Computational NeuroSurgery Lab, Macquarie University, Sydney, NSW, Australia

**Keywords:** Cerebrospinal fluid, Tryptophan-kynurenine pathway, Infantile spasms, Epileptic spasms, Metabolomics, Ketogenic diet

## Abstract

**Background:**

Epileptic (previously infantile) spasms is the most common epileptic encephalopathy occurring during infancy and is frequently associated with abnormal neurodevelopmental outcomes. Epileptic spasms have a diverse range of known (genetic, structural) and unknown aetiologies. High dose corticosteroid treatment for 4 weeks often induces remission of spasms, although the mechanism of action of corticosteroid is unclear. Animal models of epileptic spasms have shown decreased brain kynurenic acid, which is increased after treatment with the ketogenic diet. We quantified kynurenine pathway metabolites in the cerebrospinal fluid (CSF) of infants with epileptic spasms and explored clinical correlations.

**Methods:**

A panel of nine metabolites in the kynurenine pathway (tryptophan, kynurenine, kynurenic acid, 3-hydroxykynurenine, xanthurenic acid, anthranilic acid, 3-hydroxyanthranilic acid, quinolinic acid, and picolinic acid) were measured using liquid chromatography coupled to tandem mass spectrometry (LC-MS/MS). CSF collected from paediatric patients less than 3 years of age with epileptic spasms (*n*=34, 19 males, mean age 0.85, median 0.6, range 0.3–3 yrs) were compared with other epilepsy syndromes (*n*=26, 9 males, mean age 1.44, median 1.45, range 0.3–3 yrs), other non-inflammatory neurological diseases (OND) (*n*=29, 18 males, mean age 1.47, median 1.6, range 0.1–2.9 yrs) and inflammatory neurological controls (*n*=12, 4 males, mean age 1.80, median 1.80, range 0.8–2.5 yrs).

**Findings:**

There was a statistically significant decrease of CSF kynurenic acid in patients with epileptic spasms compared to OND (*p*<0.0001). In addition, the kynurenic acid/kynurenine (KYNA/KYN) ratio was lower in the epileptic spasms subgroup compared to OND (*p*<0.0001). Epileptic spasms patients who were steroid responders or partial steroid responders had lower KYNA/KYN ratio compared to patients who were refractory to steroids (*p*<0.005, *p*<0.05 respectively).

**Interpretation:**

This study demonstrates decreased CSF kynurenic acid and KYNA/KYN in epileptic spasms, which may also represent a biomarker for steroid responsiveness. Given the anti-inflammatory and neuroprotective properties of kynurenic acid, further therapeutics able to increase kynurenic acid should be explored.

**Funding:**

Financial support for the study was granted by Dale NHMRC Investigator grant APP1193648, Petre Foundation, Cerebral Palsy Alliance and Department of Biochemistry at the Children's Hospital at Westmead. Prof Guillemin is funded by NHMRC Investigator grant APP1176660 and Macquarie University.

**Research in context**

**Evidence before this study**

Epileptic (previously infantile) spasms is a common epileptic encephalopathy of early childhood and affects 0.25–0.42/1000 live births per year. Epileptic spasms present with brief and symmetric tonic seizures which can be flexor, extensor, mixed flexor or extensor, and typically occur in clusters. Spasms are typically accompanied by electrophysiological hypsarrhythmia. However, the diagnosis of epileptic spasms is often delayed, and the neuropathological mechanisms are not well understood. A few small-scale studies have reported alterations in the tryptophan-kynurenine pathway in children with epileptic disorders, including epileptic spasms. More recently, animal models and human studies have highlighted the potential role of the tryptophan-kynurenine pathway in epileptic spasms. At present, there are no disease specific treatments of epileptic spasms to improve the often-concerning neurodevelopmental outcomes.

**Added value of this study**

We present data showing changes in CSF tryptophan-kynurenine pathway metabolites in patients with epileptic spasms. Specifically, we found a significant depletion of kynurenic acid and decreased kynurenic acid/kynurenine ratio in children with epileptic spasms compared to children with other neurological conditions. The low kynurenic acid and kynurenic acid/kynurenine ratio was lower in the steroid responsive subgroups compared to the steroid refractory subgroup.

**Implications of all the available evidence**

Translatable biomarkers which define neuropathophysiologic mechanisms can improve the treatment and monitoring of epileptic spasms. The study provides evidence that the kynurenine pathway is affected in epileptic spasms, and the kynurenic acid/kynurenine ratio holds promise as a potential biomarker for epileptic spasms, and predictive response to treatment. Therapies or dietary supplements able to increase kynurenic acid levels could have neuroprotective effects and improve neurodevelopmental outcomes in children with epileptic spasms.

Alt-text: Unlabelled box

## Introduction

Epileptic spasms (previously called infantile spasms) is an early-onset epileptic encephalopathy characterised by clusters of epileptic spasms, often associated with severe developmental and neurological outcomes.^1^, ^2^, ^3^, ^4^, ^5^ Epileptic spasms is estimated to affect 1 in 5000 children^6^ with diverse structural and genetic causes,^7^, ^8^, ^9^, ^10^ however the aetiology in 40% of epileptic spasms patients remains unknown (previously termed idiopathic or cryptogenic).^11^ Improved understanding of the underlying disease mechanisms of epileptic spasms, and biomarkers to identify the underlying aetiology are of great importance.^12^

Clinical observations have reported the sites of spasm generation are a result of interactions between the cortical and brain stem neural networks.^13^^,^^14^ Positron emission tomography (PET) studies have reported significant abnormalities of hypometabolism in the cortex.^15^^,^^16^

**Figure 1:**
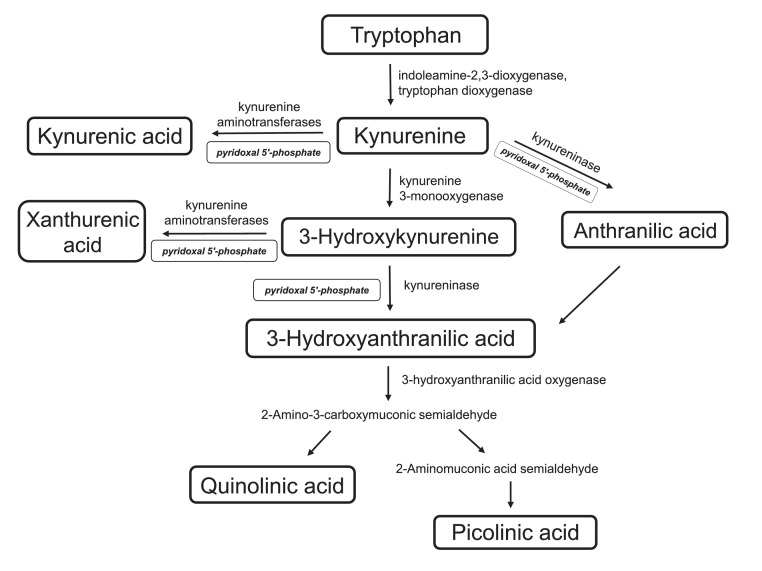
Summary of the tryptophan-kynurenine pathway including metabolites, enzymes, and vitamin cofactor where relevant.

Metabolites are crucial biomarkers of cellular function. A growing body of evidence shows the tryptophan pathway is implicated in the pathophysiology of epileptic seizures.^17^, ^18^, ^19^, ^20^, ^21^, ^22^ The kynurenine pathway (KP) is the main catabolic pathway of tryptophan (TRP) (Figure 1). Indoleamine-2,3-dioxygenase (IDO1), the first enzyme of the KP, is activated by various inflammatory mediators, and converts TRP to kynurenine (KYN). KYN is then metabolised into different branches by three main enzymes: kynurenine aminotransferase (KAT), kynurenine 3-monooxygenase (KMO) and kynureninase, leading to the formation of kynurenic acid (KYNA), 3-hydroxykynruenine (3-HK) and anthranilic acid (AA) respectively (Figure 1). 3-hydroxykynurenine is further converted to 3-hydroxyanthanthranilic acid (3-HAA) and into quinolinic acid (QUIN) by the consecutive activity of enzymes kynureninase and 3-hydroxyanthranilic acid oxygenase.^23^^,^^24^ AA can be also converted to 3-HAA through an oxidation-reduction reaction.^25^ Several intermediates in the tryptophan pathway such as KYN, KYNA and QUIN have biological involvement in neurotransmission, inflammation, immune responses and neurodegeneration, and alterations in these metabolites have been reported in various neurological diseases.^26^, ^27^, ^28^ For example, KYNA, produced by astrocytes, has anti-inflammatory and neuroprotective properties (a glutamate receptor antagonist), whereas QUIN, produced by activated microglia, is neurotoxic.^29^

A few small studies in CSF and serum of children with epileptic spasms have shown some alterations in tryptophan-kynurenine pathway metabolites, however these studies are limited by small sample sizes and heterogeneity of patients.^30^, ^31^, ^32^ Two studies by Yamamoto et al. in eight epileptic spasms patients reported decreased CSF KYNA levels and elevated 3-HK.^33^^,^^34^

In the present study, we aimed to explore the changes in CSF concentrations of metabolites in the tryptophan-kynurenine pathway using a LC-MS/MS method to compare children with epileptic spasms to controls, and explore associations with aetiology and response to treatment.

## Methods

### Study design and patient characteristics

All eligible CSF from children under 3 years of age were included in the study. The CSF neurochemistry laboratory receives ∼500 samples/year from the Children's Hospital at Westmead (CHW), and residual CSF is stored at −80 °C and available for research, subject to ethics requirements. CHW is the largest children's hospital in NSW, Australia, a state of 8 million people, and is a tertiary neurology referral centre.

#### Epileptic spasms cohort

All children had typical epileptic spasms which were either flexor or extensor spasms (or both), occurring in clusters. All children with epileptic spasms within the catchment area would be expected to be referred, and as the seizures were the first symptom of a neurological disorder, the majority of epileptic spasms patients would have CSF analysis, often at the time of MRI neuroimaging. All patients were investigated between 2010-2022 by the treating neurologists (KK, RCD, ET, SM, DG, RW, CT, JA, SAH, MM, SG) including MRI neuroimaging, CSF examination, and blood tests including genomic testing. One exception was 2 children with tuberous sclerosis complex who presented during the study period- neither had CSF examination as the preceding skin examination and MRI brain was suggestive of TSC, and CSF examination was not deemed required. The epileptic spasms patients (*n*=34, 19 males, mean age 0.85, median 0.6, range 0.3–3 yrs) were phenotyped from the electronic medical records by RCD, blinded to the CSF metabolic data. CSF was taken mean 6.7 weeks after historical spasm onset (range 2 days -26 weeks) (supplementary Table 1). At the time of CSF sampling, 5 patients were taking an anti-epileptic (vigabatrin, benzodiazepine, leviteracetam, carbamazepine), but no patients had received corticosteroid. Clinical data including response to corticosteroid treatment was recorded (responder, partial responder, refractory).^3^ Steroid responders were patients who had complete spasms remission rapidly within 10 days of steroid monotherapy. Partial responders were patients who had clear reduction in spasms, but reduction was incomplete, or complete but was associated with the use of other drugs (benzodiazepines or vigabatrin) so that inferring steroid responsiveness was less definitive. Steroid refractory patients did not have meaningful spasm reduction with the use of steroids, either as monotherapy or combined therapy.

Aetiological classification was performed blinded to the CSF data by the treating clinicians, as per International League Against Epilepsy (ILAE) with known aetiology (genetic, structural, etc) and unknown aetiology.^35^, ^36^, ^37^ Further phenotypic data is available in Supplementary Table 1.

#### Controls

As the epileptic spasm group were between age 0.3 and 3 years, for comparative data, all controls who were younger than 3 years of age and had their CSF during the same time period as the epileptic spasms group, were included subject to ethics requirements. It is ethically impossible and inappropriate to gather CSF from healthy normal children therefore we used the following control groups who had CSF testing to investigate their neurological condition:

1. Neuroinflammatory control (NI) group (*n*=12, 4 males, mean age 1.80, median 1.80, range 0.8–2.7 yrs) was used as a positive control group to define CSF metabolites in definite neuroinflammatory conditions including encephalitis (*n*=7 [2 enteroviral, 1 influenza, 1 Human Herpes Virus 6, 3 unknown cause], cerebellitis, opsoclonus myoclonus ataxia syndrome, pneumococcal meningoencephalitis, Aicardi Goutières syndrome *SAMHD1* mutation (*n*=1 each) (Supplementary Table 2).
2. Other Non-inflammatory neurological group (OND) (*n*=29, 18 males, mean age 1.48, median 1.6, range 0.1–3.0 yrs) was used as the normative data (negative control). These patients had neurological conditions which did not include epilepsy and were non-inflammatory in origin including neurogenetic causes (*n*=15), structural causes such as cerebral palsy (*n*= 7), neurodevelopmental disorders such as autism (*n*=7) (detailed in Supplementary Table 3).
3. Other epilepsy patients (*n*=26, 9 males, mean age 1.44, median 1.45, range 0.3–3 yrs) were used to determine differences in the CSF metabolites of the epileptic spasms group compared to other seizures/epilepsy. This control group included patients with current active seizures but no spasms, including recent status epilepticus (*n*=7 [febrile status epilepticus *n*=5, afebrile status epilepticus *n*=2]), Developmental Epileptic Encephalopathy (DEE, *n*=11 [due to genomic variants in *SCN8A n*=3, *SCN2A n*=2, *GRIN1, GLUT1, STXBP1, FOXG1,* Angelman syndrome, no gene found all *n*=1]), suspected genetic epilepsy but no cause found (*n*=5), symptomatic epilepsy (*n*=3 [epilepsy associated with focal cortical dysplasia *n*=2, and epilepsy due to cerebral palsy *n*=1] (Supplementary Table 4).

### Tryptophan-kynurenine metabolites quantification

CSF were collected into five tubes using an aseptic technique and frozen within 1 hour of sampling and stored at –80 °C until the time of processing. The CSF tube analysed for this study was not used for routine testing and thawed only once after sampling. The tryptophan-kynurenine pathway metabolites (TRP, KYN, KYNA, 3-HK, XAN, AA, 3-HAA, QUIN, and picolinic acid) were measured according to the method of Yan et al.^38^ Briefly, 80 μL of human CSF was deproteinised with 20 μL of metaphosphoric acid/ethylenediaminetetraacetic acid solution, vortexed and centrifuged in Nanosep 0.2 μM centrifugal devices. The collected supernatant was analysed using a Waters ACQUITY UPLC I-Class System UPLC system coupled to a Xevo TQ-XS triple quadrupole mass spectrometer. The separation of metabolites was carried out using a twelve minute gradient programme on the Acquity UPLC BEH C18 column (2.1 mm × 150 mm 1.7 μm particle size). The metabolites were detected on the mass spectrometer in the multiple reaction monitoring mode (MRM) using positive electrospray ionisation.

### Multiplex cytokine/chemokine assay

Thirty-two cytokines were measured by multiplexed fluorescent bead-based immunoassay detection (MILLIPLEX® MAP system, Millipore Corporation, Missouri U.S.A.) according to the manufacturer's instructions, using a combination of 23-plex (MPHCYTOMAG60K23), 6-plex (MPHCYP2MAG62K06), and 3-plex (MPHCYP3MAG63K03) Millipore Human Cytokine panel kits. The 23-plex kit contained antibody-conjugated beads for IL-1Ra, GM-CSF, IL-1β, TNF-α, IL-2, IL-4, IL-6, IL-8, IL-10, IL-13, IL-17A, IFN‐γ, CCL2/MCP-1, CCL5/RANTES, CXCL1/GRO, CXCL10/IP-10, CCL3/MIP-1α, CCL4/MIP-1β, IL-12p40 and IL-12p70, IFN-α2, G-CSF and CCL11/Eotaxin. The 6-plex kit was used to detect IL-21, IL-23, CXCL13/BCA-1, CCL17/TARC, CCL21/6Ckine and CXCL12/SDF-1. The 3-plex kit contained antibody-conjugated beads for CXCL9/MIG, CXCL11/I-TAC, and CCL19/MIP-3β. Adequate CSF was not available for all of the patients and controls, but included epileptic spasms (21/34), OND (20/29), other seizures/epilepsy (21/26) and neuroinflammatory (12/12) controls. The same method was used as described previously.^39^

### Measurement of pyridoxal 5′-phosphate (PLP)

As pyridoxal 5’-phosphate (PLP) is a cofactor of enzymes in the KP (Figure 1), we quantified PLP. Protein precipitation was carried out by mixing 120 μL of CSF with an equal volume of 0.3 N trichloroacetic acid solution in Nanosep with 3K omega centrifugal devices. D_5_-pyridoxal 5’-phosphate was added to the precipitation reagent, trichloroacetic acid solution. The CSF samples were vortexed for 60 s, precipitated on ice in the dark at 5 °C for 30 min and centrifuged at 12,000 g for 12 min at 5 °C. Quantification of pyridoxal 5′-phosphate was performed using the Acquity Premier BEH C18 column (2.1 mm × 150 mm 1.7 μm particle size) on the Waters ACQUITY UPLC I-Class System UPLC system coupled to a Xevo TQ-XS triple quadrupole mass spectrometer. The mobile phases consisted of 0.1% formic acid in water (A) and 0.1% formic acid in acetonitrile (B) was run at a flow rate of 0.20 mL min**^−1^** and an injection volume of 5 μL. The gradient programme was: 0 to 1 min (2.5 % B), 1 to 7.5 min (2.5–50% B), 7.5 to 8.5 min (50-1 % B), 8.5 to 9 min (1–2.5% B) and 9 to 12 min (2.5% B).

The mass spectrometer was operated in the MRM mode using positive electrospray ionisation. The source conditions were as follows: source temperature of 150 °C, desolvation temperature was 500 °C, nitrogen gas flow rate of 600 L h**^−1^**, cone voltage 20 V and capillary voltage of 2.50 kV. The MRM transitions monitored were m/z 248 → 150 (16 eV collision energy, 30 cone voltage) for pyridoxal 5’-phosphate. The dwell time for each transition was 300 ms and retention times for pyridoxal 5’-phosphate was at 4.08 min.

### Statistics

**Figure 2:**
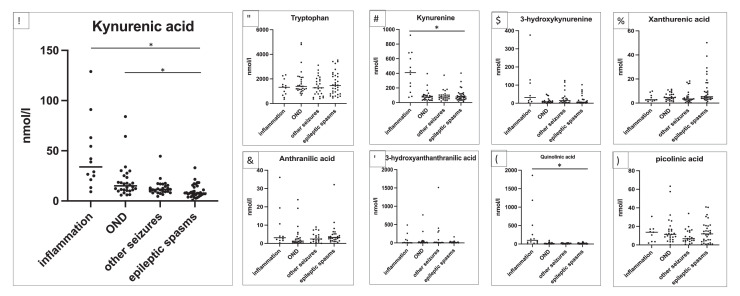
Quantitative data in nmol L^−1^ of individual CSF patient samples for tryptophan-kynurenine metabolites in neuroinflammatory control group (*n*=12), other non-inflammatory neurological controls (OND, *n*=29), other seizures (*n*=26) and epileptic spasms (*n*=34). The primary group of interest was the epileptic spasms group, so pairwise comparisons with Mann Whitney U test were performed between the epileptic spasms group and the 3 control groups and are presented in supplementary table 6. Only statistically significant findings after Bonferroni correction are presented in the figure. Kynurenic acid was statistically significantly decreased in epileptic spasms compared to neuroinflammatory controls and OND (both *p*<0.0001) (Mann Whitney U test) (Figure 2a). As expected, the neuroinflammatory group had higher kynurenine (Figure 2c) and quinolinic acid (Figure 2h) compared to epileptic spasms (both *p*<0.0001) (Mann Whitney U test). Asterisks above the horizontal lines of the groups indicate significant difference *p*<0.0001.

**Figure 3:**
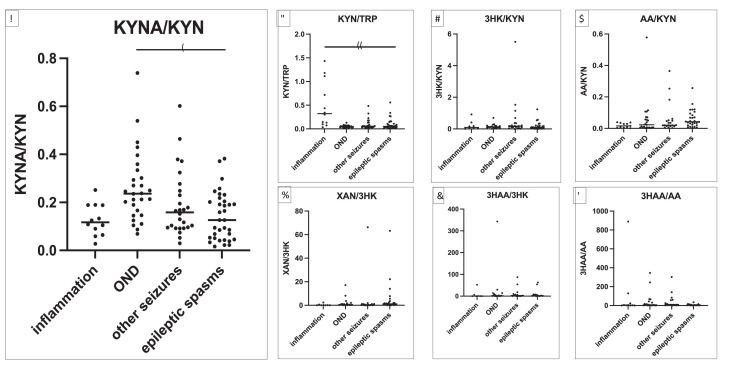
Adjacent ratios in patients with neuroinflammatory control group (*n*=12), other non-inflammatory neurological controls (OND, *n*=29), other seizures (*n*=26) and epileptic (epileptic) spasms (*n*=34). The primary group of interest was the epileptic spasms group, so pairwise comparisons with Mann Whitney U test were performed between the epileptic spasms group and the 3 control groups, and are presented in supplementary table 7. Only statistically significant findings after Bonferroni correction are presented in the figure. A decrease of the KYNA/KYN ratio was observed in the epileptic spasms group compared to OND controls (*p*=0.0002) (Mann Whitney U test) (Figure 3a). As expected, the KYN/TRP ratio was higher in the neuroinflammatory group compared to the epileptic spasms group (*p*=0.0001) (Mann Whitney U test) (Figure 3b). Asterisks above the horizontal lines of the groups indicate significant difference: *p=0.0002 and **p=0.0001.

Statistical analyses were performed using SPSS version 26 (IBM Corp. Armonk, NY, USA) and graphs were generated by GraphPad Prism 8 (GraphPad, San Diego, USA). As the metabolomic data was not normally distributed, non-parametric statistics (Mann–Whitney U test) was used for analysis. The primary hypothesis was that patients with epileptic spasms have significantly different kynurenine metabolites compared to the control groups. Therefore, pairwise comparisons between the epileptic spasms group and the 3 control groups were performed for the 9 metabolites (Figure 2) and the ratios (Figure 3), and given the multiple pairwise testing, Bonferroni corrections were made.

Analysis of correlations between metabolites in epileptic spasms was performed using Spearman's correlation coefficient (R) in SPSS. The correlations are presented in colour (red for positive correlation, green for negative correlation). The strength of the statistically significant correlations (Spearman's Rs) was also graphically generated in Xmind (https://www.xmind.net/download/).

### Ethics

The Sydney Children's Hospitals Network Ethics Committee approved this study (LNR/14/SCHN/275; 2019/ETH06182), including informed consent from parents and/or guardians, as per ethics protocol.

### Role of funders

Financial support for the study was granted by Dale NHMRC Investigator grant APP1193648, Petre Foundation and Cerebral Palsy Alliance. Funding sources had no role in the design of this study, and did not have any role during its execution, analyses, interpretation of the data, or decision to submit results.

## Results

### Epileptic spasms cohort

In the epileptic spasms cohort (*n*=34, 19 males, mean age 0.85, median 0.6, range 0.3–3 yrs), acute electroencephalography showed hypsarrhythmia (*n*=8), modified hypsarrhythmia (*n*=19), and epileptic EEG without hypsarrhythmia (*n*=7). In order to classify the aetiology, brain MRI was done in all 34 patients, and was normal (*n*=20) or showed focal cortical dysplasia (FCD), other malformation, atrophy and brain injury (*n*=14) (Table 1 and Supplementary Table 1). Twenty-eight patients had a comparative genomic hybridization microarray which was diagnostic in 4, and 12 had an epileptic gene panel which was diagnostic in 3. After investigation and follow-up (median 2.5 years range 0.1–9.5), the treating clinician aetiological diagnoses (Table 1) included known aetiology (*n*=19 in total, including genetic *n*=8 and structural *n*=11 [8 FCD and 3 acquired brain injury]), and unknown aetiology (*n*=15) (10 would have previously been termed idiopathic and 5 would have previously been termed cryptogenic) (Table 1 and Supplementary Table 1).

**Table 1:** Epileptic spasm subgroups according to aetiology including age at CSF sampling, neuroimaging, follow-up duration and outcomes. Individual data available in Supplementary Table 1.

Aetiology	Med. age, range	Detailed aetiology	Follow-up, med. (range)	Ongoing epilepsy % a	Developmental outcomes (%)				
					Norm.	Mild DD	Mod-sev DD	ASD	CP
Genetic (*n*=8)	0.95 (0.2–1.6)	*CDKL5, FOXG1, SCN2A (all heterozygote), TBCK* (homozygous), Trisomy 21, 16p13.11 microdeletion, 14q12 dup including *FOXG1*, progressive leukoencephalopathy (all *n*=1).	2.75 (2–14)	62.5	0	12.5	87.5	25	50
Structural (*n*=11)	0.7 (0.5–1.5)	FCD (*n*=6), hemispheric mal. (*n*=2), brain injury due to hypoglycaemia, HSVE and HIE (all *n*=1)	2.5 (0.3–8.5)	73	9	27	64	9	45
Unknown (*n*=15)	0.51 (0.25–0.8)	Unknown	2.87 (0.1–9.5)	20^b^	13	47	33	13	0

a Ongoing seizures or ongoing anti-epileptic treatment.

b one patient had no follow-up (left country). ASD: autistic spectrum disorder, CP: cerebral palsy, DD: developmental delay, FCD: focal cortical dysplasia, HSVE: Herps simplex virus encephalitis, mal.: malformation, med.: median, mod-sev: moderate-severe.

United Kingdom infantile spasms study (UKISS) prednisolone protocol was given in 31/34, vigabatrin to 16/34, and 16/34 (47%) are on ongoing anti-epileptic drugs (AED) treatment at median 2.5 years follow-up. No patients received ACTH. Patients were defined as steroid responder (*n*=7), partial responder (*n*=18), and refractory (*n*=6) based on response to the initial UKISS prednisolone regimen (*n*=31 treated). Only 3 of 34 patients had normal neurodevelopmental outcomes, although the unknown aetiology subgroup had generally less epilepsy and less severe neurodevelopmental outcomes than the other 2 subgroups (Table 1 and Supplementary Table 1). The CSF sampling for the patients were conducted as part of the initial investigation, prior to treatment, and hence represents a ‘baseline’ assessment for metabolomics analysis (timing of CSF sampling relative to spasm onset is presented in supplementary Table 1).

There was no statistically significant difference in the frequencies of male and female patients between the epileptic spasms group (*n*=34) and the three control groups. The epileptic spasms patients were statistically younger than the OND controls (*p*<0.001) (Mann Whitney U test). Therefore, a sub-analysis of patients less than one year of age was also performed between the epileptic spasms and OND groups (see later) and there was no statistical difference in age (*p*=0.778) (Mann Whitney U test).

### CSF metabolomics

Nine metabolites in the KP were measured in epileptic spasms patients and the three control groups (Figure 2). There were five metabolites (3-HK, xanthurenic acid, AA, 3-HAA and picolinic acid) that had a small percentage of samples below the lower detection limit (Supplementary Table 5). 3 pairwise comparisons were made for the 9 metabolites, therefore Bonferroni correction was performed (all comparisons presented in Supplementary Table 6). As expected, kynurenic acid, kynurenine and quinolinic acid were higher in the neuroinflammation group than the epileptic spasms (all <0.0001) (Mann Whitney U test) (Figure 2). KYNA was significantly decreased in the epileptic spasms group compared to OND (*p*<0.0001) (Mann Whitney U test) (Figure 2).

Sub-analysis of patients less than one year of age in epileptic spasms (*n*=26, mean age 0.55yr, median age 0.5yr) and OND (*n*=17, mean age 0.49 yr, median age 0.4 yr) retained the statistical differences for KYNA (lower in epileptic spasms, *p*=0.0003) (Mann Whitney U test) (Supplementary Figure 1).

Ratios are often used as representative markers of enzymatic activity (Figure 1). 3 pairwise comparisons were made for the 7 ratios, therefore Bonferroni correction was performed (all comparisons presented in Supplementary Table 7). The kynurenine/tryptophan ratio (KYN/TRP) is a marker of IDO1 and tryptophan 2,3-dioxygenase (TDO) enzymatic activity (IDO1 is activated by cytokines). KYN/TRP was not different in the epileptic spasms group compared to the OND group, but was lower in epileptic spasms compared to the neuroinflammatory group as expected (Figure 3). By contrast, the kynurenic acid/kynurenine ratio (KYNA/KYN), a marker of KAT enzymatic activity was significantly decreased in the epileptic spasms group compared to the OND group (*p*=0.0002) (Mann Whitney U test) (Figure 3). KYNA/KYN ratio remained statistically lower in epileptic spasms group compared to OND group when only patients under 1 year were included (*p*=0.0037) (Mann Whitney U test) (Supplementary Figure 1).

**Figure 4:**
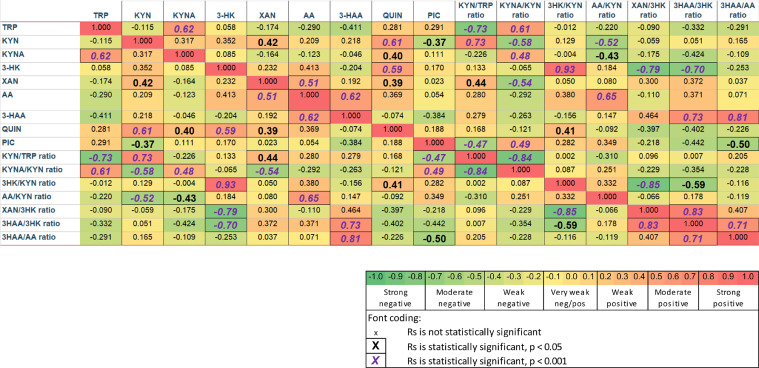
Spearmen's R correlations between metabolites in epileptic spasms. The correlations emphasise the described findings in Figure 2, Figure 3, but also uncover moderate positive correlation in epileptic spasms between quinolinic acid with kynurenine and 3-HK. In addition, there is a positive correlation between TRP and KYNA. There is a weaker positive correlation of quinolinic acid with KYNA and XAN.

To understand interactions between metabolites, Spearman's Rs were calculated and presented in Figure 4 for the epileptic spasms group. The correlations emphasised the main findings as above, but also uncovered moderate positive correlations in epileptic spasms of QUIN with KYN and 3-HK, and weaker positive correlations of QUIN with KYNA and XAN. An additional uncovered positive correlation in the epileptic spasms group was between TRP and KYNA (Figure 4) (graphically presented in XMind in Supplementary Figure 2).

In order to examine for active inflammation, 32 cytokines/chemokines were measured using multiplex panel, and the neuroinflammatory group had elevated cytokines/chemokines, as expected (Supplementary Figure 3). Only CXCL12 was significantly different in epileptic spasms compared to OND group (decreased, *p*<0.0001) (Mann Whitney U test). No other cytokines/chemokines were significantly different in epileptic spasms group compared to OND group (Supplementary Figure 3).

**Figure 5:**
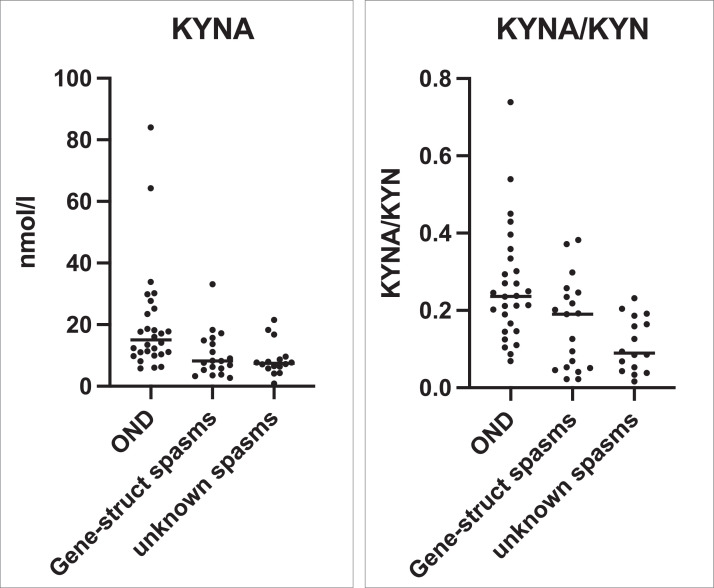
The primary findings from Figures 2 and 3 (KYNA and KYNA/KYN) are examined for aetiological associations in epileptic spasms. Comparison of CSF KYNA and KYNA/KYN ratio in the unknown aetiology epileptic spasms subgroup (*n*=15) with the known aetiology (genetic-structural) epileptic spasms subgroup (*n*=19), and statistically compared using the Mann-Whitney U test. Other non-inflammatory neurological controls (OND, *n*=29) are presented for comparison, but are not included in statistical testing. KYNA was not lower in the unknown aetiology subgroup compared to the gene-structural subgroup (*p*=0.838) (Mann Whitney U test). Although KYNA/KYN was lower in unknown aetiology subgroup compared to gene-structural subgroup, the finding was not statistically significant (*p*=0.164) (Mann Whitney U test).

To determine if the KP variations were common to all epileptic spasms patients, or specific to the unknown aetiology epileptic spasms subgroup, we compared KYNA and KYNA/KYN in the unknown aetiology IS subgroup compared to the known aetiology epileptic spasms subgroup (genetic-structural), but there was no significant difference (Figure 5).

**Figure 6:**
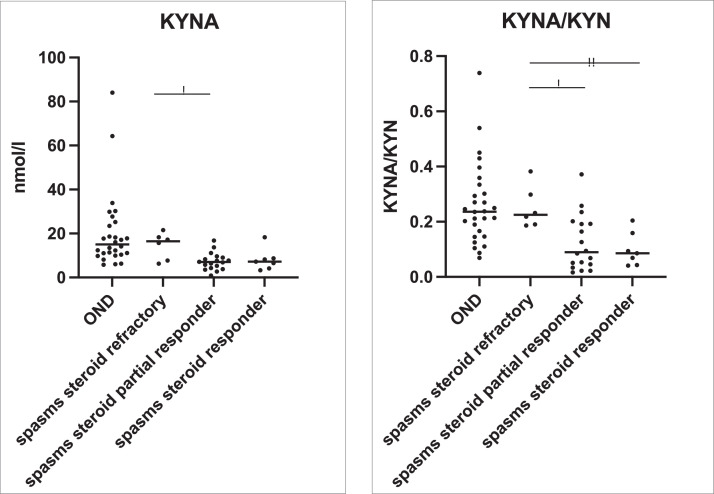
The primary findings from Figures 2 and 3 (KYNA and KYNA/KYN) are examined for steroid responsiveness in epileptic spasms. KYNA and KYNA/KYN ratio are presented in steroid responders (*n*=7), partial steroid responders (*n*=18) and steroid refractory (*n*=6) patients. OND group (*n*=29) are presented for comparison, but not subjected to statistical testing. KYNA is lower in the steroid partial responders compared to the steroid refractory patients (*p*=0.0183) (Mann Whitney U test). KYNA/KYN ratio is lower in the steroid partial responder group compared to the steroid refractory group (*p*=0.0183) (Mann-Whitney U test), and KYNA/KYN is lower in the steroid responder group compared to the steroid refractory group (*p*=0.0047) (Mann-Whitney U test). The comparison of groups were analysed by the Mann-Whitney U test, and uncorrected *p* values are presented.

The epileptic spasms patients who were partial steroid responders had lower KYNA compared to the steroid refractory patients (*p*=0.0183) (Mann Whitney U test) (Figure 6). The epileptic spasms patients who were steroid responders and partial responders had lower KYNA/KYN ratio than the steroid refractory patients (*p*<0.005, *p*<0.05 respectively) (Mann-Whitney U test) (Figure 6). The analysis of epileptic spasms subgroups showed a clear trend of lower KYNA/KYN ratio associated with steroid responsiveness in both the known aetiology IS group, and the unknown epileptic spasms group (Supplementary Figure 4).

Pyridoxine 5 phosphate (PLP) showed no significant differences in the tested epileptic spasms patients compared to OND, and there was no correlation of KYNA or KYNA/KYN with PLP in the tested epileptic spasms patients (Supplementary Figure 5).

## Discussion

We investigated the levels of CSF KP metabolites in a cohort of epileptic spasms. The main limitation of our cohort was the absence of tuberous sclerosis complex (TSC) which is described in 5.7% of a large Chinese epileptic spasms cohort (*n*=541)^36^ and 7.2% of a UK cohort (*n*=207).^35^ Two patients presented with spasms due to TSC during our study period, but they did not have CSF examination, possibly because TSC is often diagnosed on skin examination and MRI neuroimaging, and therefore CSF examination was not considered necessary. Other than TSC, our epileptic spasms cohort was representative of the most tertiary epileptic spasms cohorts in proportions of known and unknown aetiology.

In the present study, we report a significantly lower KYNA in epileptic spasms compared to OND. The direct precursor-product ratio is commonly used to infer enzymatic activity, and we demonstrate statistically unchanged KYN/TRP ratio, but a significantly decreased KYNA/KYN ratio in epileptic spasms compared to other non-inflammatory neurological controls. The KYNA/KYN ratio was lower in all epileptic spasms subgroups, both known and unknown aetiology.

Previous studies have shown that KYNA has neuroprotective, anticonvulsant and anti-inflammatory properties,^40^^,^^41^ and acts as an endogenous antagonist of the N-methyl-D aspartate and α7 nicotinic acetylcholine receptors, blocking excitotoxic insults and apoptotic processes.^40^, ^41^, ^42^ Therefore, a decreased KYNA could result in a pro-convulsant and neurotoxic state. There are very few CSF studies in epileptic spasms, and our results are in line with the current small literature showing decreased concentrations of CSF KYNA.^30^^,^^31^^,^^34^ KYNA does not cross the blood-brain barrier due to its polar structure and is mostly produced by astrocytes by the KAT enzymes.^23^ It has been reported that a decrease in the KYNA/KYN ratio is associated with reduction of KAT enzyme activity.^43^^,^^44^ Vitamin B6 in the form of PLP is a cofactor that influences the catabolism of TRP (Figure 1).^45^^,^^46^ As PLP is a cofactor for KAT activity (which metabolises KYN to KYNA), we measured PLP in a subgroup of patients and controls but found no evidence of PLP deficiency in the CSF of children with epileptic spasms, and no correlation with KYNA/KYN ratio. We hypothesised that, as in the rodent model of epileptic spasms,^47^ there could be IDO1 activation due to inflammation. However, we could find little evidence of current active neuroinflammation using recognised immune biomarkers such as cytokines and chemokines. Therefore, the biological trigger activating and dysregulating the KP in epileptic spasms is still unknown, although preceding neuroinflammation and/or microbiome dysbiosis (see below) remain plausible.

The current recommended standard first-line treatments for epileptic spasms are adrenocorticotropic hormone or oral corticosteroids and/or vigabatrin.^1^^,^^3^^,^^4^^,^^48^ There is strong evidence that high dose corticosteroids is highly effective in inducing spasm remission, and the UKISS protocol now recommends oral prednisolone, starting at 10 mg four times per day.^3^ It is our practise to adopt the UKISS prednisolone protocol and we do not use ACTH any longer. Previously, it was perceived that corticosteroids were more effective in idiopathic or cryptogenic cases (now collectively called unknown aetiology), whereas vigabatrin was more effective in symptomatic cases, including TSC complex. A recent RCT showed that combining both oral prednisolone and vigabatrin is superior to oral prednisolone alone in inducing spasm remission.^3^ Although delayed treatment can worsen neurological outcomes, the use of combined corticosteroid plus vigabatrin does not improve long term outcomes compared to prednisolone alone.^5^ At present, it seems the long-term outcomes are predominantly dictated by the underlying aetiology, and there are no ‘disease modifying medications’ that alter long term trajectories for epileptic spasms, to date.

The ketogenic diet (KD) is a high-fat, low-carbohydrate, and adequate-protein diet also used to treat seizures including epileptic spasms, with international best practice guidelines recommending consideration of the diet early in treatment course of epileptic spasms.^49^^,^^50^ Interestingly, a recent study reported that the ketogenic diet is equally as effective as adrenocorticotropic hormone treatment for epileptic spasms, and with less adverse effects.^51^ More recent evidence has demonstrated that the KD affects plasma KYN metabolites in children with refractory epilepsy,^52^ and the KD resulted an increase in KYNA levels, and significantly increase in the KYNA/KYN plasma ratio.^52^ An animal model of brain injury with refractory epileptic spasms (intractable to conventional anti-convulsants), showed reduced hippocampal KYNA, which was normalised following KD treatments and associated with the remission of epileptic spasms.^47^ Mu et al. demonstrated that antibiotics (minocycline, an IDO1 inhibitor) also reduced spasms, increased the effectiveness of the ketogenic diet, and elevated hippocampal kynurenic acid.^47^ This spasms animal model showed that the microbiome was a key mediator of this effect, as transplanting faeces from animals treated with KD into animals with ongoing epileptic spasms was effective in reducing the seizures. These prior observations, in conjunction with our findings, support the concept that the downregulation of KYNA may be a key player in the pathogenesis of epileptic spasms, and therapeutics that increase KYNA could be neuroprotective and improve epilepsy and neurodevelopmental outcomes in epileptic spasms.

In our cohort, the unknown aetiology epileptic spasms subgroup generally responded rapidly to steroids, and had better epilepsy and neurodevelopmental outcomes than patients with known aetiologies (Table 1 and supplementary Table 1), as previous described.^53^, ^54^, ^55^ Although the neurodevelopmental outcomes were less severe in the unknown aetiology subgroup, only two of 15 unknown aetiology epileptic spasms children were considered normal at follow-up, and the follow-up duration was too short to confidently detect milder learning issues. In our study, the KYNA/KYN ratio was low in all epileptic spasms subgroups. However, the children who responded to steroids fully or partially had lower KYNA/KYN ratio than patients who were refractory to steroids. Therefore, we propose that the KYNA/KYN ratio holds promise as a potential biomarker of epileptic spasms, and also response to steroid therapy.

There was an elevation of anthranilic acid and xanthurenic acid in the epileptic spasms cohort, although these findings were not significant after Bonferroni correction (Supplementary Table 6). However, these metabolites could be worthy of future research in this context.

Due to the neuroprotective and anti-inflammatory properties of KYNA, there has been interest in supplementing TRP or KYNA in some neurological diseases, including emerging clinical trials targeting the KP in neurological diseases such as chronic pain and multiple sclerosis.^56^ As TRP is predominantly sourced from food and gut metabolism, nutritional therapeutics or microbiome manipulation has been a focus of attention. There is interest in foods enriched with KYNA, such as breast milk (20 times more KYNA than formula milk), honey, or broccoli.^57^^,^^58^ However, KYNA is not permeable across the blood-brain barrier, although its precursor L-Kynurenine sulfate is permeable, and could be a therapeutic route to elevate brain KYNA concentrations.^28^ Given the fact the KD elevates KYNA, the application of KD in epileptic spasms warrants further attention. We believe that the positive correlation between TRP and KYNA (Figure 4) in the epileptic spasms patients supports the hypothesis that dietary modification of TRP may optimise KYNA levels.

Finally, given that KYNA is a glutamate antagonist, we hypothesise that alternative glutamate antagonists such as amantadine could be useful, as has been used empirically in some epilepsies.^59^ Additionally, clinically approved glutamate receptor antagonists such as felbamate,^60^ topiramate,^61^ phenobarbital^62^^,^^63^ and perampanel^64^ have been reported to reduce seizures and show some potential as therapeutic approaches to epileptic syndromes including epileptic spasms. In addition, studies have demonstrated memantine significantly improves cognition^65^ and neurodevelopmental outcomes in epileptic patients.^66^

### Limitations

Firstly, our metabolite panel was limited to the KP, and there may be other unidentified metabolite biomarkers of epileptic spasms. Future untargeted metabolomics and proteomics studies in epileptic spasms may identify new discriminative biomarkers.

Secondly, future studies should perform combined analysis of CSF, blood and urine (and stool) samples in order to identify the origins of these metabolite changes. The less invasive and accessible nature of blood and urine metabolic markers would be beneficial in monitoring response to treatments. Further studies on pre and post steroid treatment for KP metabolite comparisons are required to understand the CSF changes in responders and non-responders to treatments.

Thirdly, not all of the children in our cohort had comprehensive genetic studies, and we may have missed some children with an underlying genetic aetiology. A further limitation was that our study was conducted in a retrospective manner, resulting in a non-standardised mode of investigation, treatment and follow-up. However, the follow-up and aetiology findings over time were accessible and documented by the treating neurologists who performed clinical phenotyping and aetiological associations independently of the laboratory metabolomic analysis. The documentation on the timing of presentation, CSF collections relative to spasm onset, and drugs given at the time of CSF sampling are important factors that can influence the KP.

Fourthly, the subgroups in the epileptic spasms cohort were of modest size, which resulted in potential statistical power problems in analysis of the subgroups. And as discussed above, although we believe our cohort is comparable to large aetiological cohorts in terms of genetic, symptomatic and unknown subgroups,^35^^,^^36^ the absence of TSC was a limitation of our cohort.

Finally, our study also did not examine microbiome characteristics or metabolism, which is a limitation given the recent animal models implicating the microbiome in reducing epileptic spasms.^47^

## Conclusion

Early diagnosis of epileptic spasms is of paramount importance to ensure rapid treatments and improved clinical care. We provide evidence that kynurenic acid and the kynurenine metabolism is altered in epileptic spasms, which supports animal model evidence of the role of the KP in epileptic spasms. Our findings support the concept that therapeutics that effect KP, such as ketogenic diet, could have neuroprotective effects and improve neurodevelopmental outcomes. Future research in combined tissue (CSF/blood/urine/stool) analysis, and untargeted metabolomics to identify novel biomarkers are required.

## Contributors

**J. Yan:** Conceptualization, Investigation, Methodology, Formal Analysis, Validation, Writing - original draft, Writing – reviewing & editing. **K. Kothur:** Investigation (cytokines), Formal Analysis (cytokines), Validation, Writing – reviewing & editing. **E.A. Innes:** Writing – Validation, reviewing & editing. **V.X. Han:** Validation (phenotyping patients from electronic medical record), Writing – reviewing & editing. **H.F. Jones:** Validation (phenotyping patients from electronic medical record), Writing – reviewing & editing. **S. Patel:** Validation (creation of redcap database for phenotyping), Writing – reviewing & editing. **E. Tsang:** Validation (ketogenic diet expertise and patient recruitment), Writing – reviewing & editing. **R. Webster:** Validation, Writing – reviewing & editing. **S. Gupta:** Validation, Writing – reviewing & editing. **C. Troedson:** Validation, Writing – reviewing & editing. **M.P. Menezes:** Validation, Writing – reviewing & editing. **J. Antony:** Validation, Writing – reviewing & editing. **S. Ardern-Holmes:** Validation, Writing – reviewing & editing. **E. Tantsis:** Validation, Writing – reviewing & editing. **S. Mohammad:** Validation, Writing – reviewing & editing. **L. Wienholt:** Investigation (cytokines), Formal Analysis (cytokines), Writing – reviewing & editing. **A.S. Pires:** Validation (kynurenine scientific discussions and hypotheses), Writing – reviewing & editing. **B. Heng:** Validation (kynurenine scientific discussions and hypotheses), Writing – reviewing & editing. **G.J. Guillemin:** Validation (kynurenine scientific discussions and hypotheses), Writing – reviewing & editing. **A. Guller:** Validation (statistical correlation analysis), Writing – reviewing & editing. **D. Gill:** Validation, Writing – reviewing & editing. **S. Bandodkar:** Validation, Verification of underlying data, Supervision, Funding acquisition, Writing – reviewing & editing. **R.C. Dale:** Conceptualization, Investigation, Validation, Verification of underlying data, Supervision, Funding acquisition, Writing - original draft, Writing – reviewing & editing. JJY, SB and RCD have directly accessed and verified the underlying data reported in the manuscript. All authors have read and approved the final version of the manuscript.

## Data sharing statement

The data supporting the findings of this study and de-identified individual participant data are available in the article and/or supplementary material. Readers are welcome to contact the corresponding author for the raw data used in this work.

## Declaration of interests

DG has received honoraria for speaking from UCB, BioMarin and Eisai. The other authors have declared that no conflict of interest exists.

## Appendix Supplementary materials

**Supplementary Figure 1.** Analysis of patients under one year of age with OND (n=17, mean age 0.49 yr, median age 0.4 yr) and epileptic spasms (n=26, mean age 0.55yr, median age 0.5yr) showed age was not a factor driving the KYNA of KYNA/KYN changes in epileptic spasms. KYNA and KYNA/KYN ratio remained statistically lower in epileptic spasms group compared to OND group when only patients under 1 year were included (p=0.0003 (*) and p=0.0037 (**), respectively) (Mann Whitney U test). Image, application 1

**Supplementary Figure 2:** Examining the direction and the strength of the statistically significant correlations (Spearman's Rs) in the epileptic spasms group (ES), created in Xmind (https://www.xmind.net/download/). The stronger the correlation, the shorter the line is. Red lines show positive correlations, blue line shows negative correlation. Moderate strong positive correlations have Rs from 0.50 to 0.79, and weak positive correlations have Rs from 0.20 to 0.49. The most “connected” metabolite is QUIN which has multiple positive loops, with 2 stronger positive correlations (3-HK and KYN) and two weaker positive correlations (XAN and KYNA). KYNA has a moderate strong positive correlation with TRP. There are no correlations between TRP and KP pathway metabolites, other than KYNA. We hypothesise that this implies that by increasing the level of TRP (e.g., via diet), it is possible to increase the level of KYNA. There is weak negative correlation between KYN and PIC. Image, application 2

**Supplementary Figure 3.** 32 cytokines and chemokines are presented in neuroinflammatory control group (n=19, 7 extra patients under 3 years old with encephalitis included for power), other non-inflammatory neurological controls (OND, n=20), other seizures (n=21) and epileptic spasms (n=21). The inflammatory neurological group had elevated cytokines/chemokines as expected. Cytokines and chemokines in epileptic spasms were compared with OND using pairwise Mann Whitney U test. Cytokines/chemokines were not statistically different in epileptic spasms compared to OND, apart from CXCL12 which was decreased in epileptic spasms compared to OND (p<0.0001) (Mann Whitney U test). Image, application 3

**Supplementary Figure 4.** The analysis of epileptic spasms subgroups showed a clear trend of lower KYNA/KYN ratio associated with steroid responsiveness in both the known aetiology epileptic spasms group (gene-structural), and the unknown aetiology epileptic spasms group. Image, application 4

**Supplementary Figure 5.** A comparison CSF pyridoxal 5’-phosphate (PLP) in epileptic spasms (n=5) and other non-inflammatory neurological controls (n=5) showed that PLP is not deficient in tested epileptic spasms patients, and the PLP concentration did not differ significantly in epileptic spasms compared to controls. Correlations of kynurenic acid and kynurenic acid/kynurenine ratio with pyridoxal 5’-phosphate revealed no statistical correlation. Image, application 5

Image, application 6
