# Supplementary figures and images for: Decreased cerebrospinal fluid kynurenic acid in epileptic spasms: A biomarker of response to corticosteroids
Source: eBioMedicine. 2022 Sep 26;84:104280. doi: 10.1016/j.ebiom.2022.104280 (PMC9515432; doi:10.1016/j.ebiom.2022.104280)

# KYNA <1yr

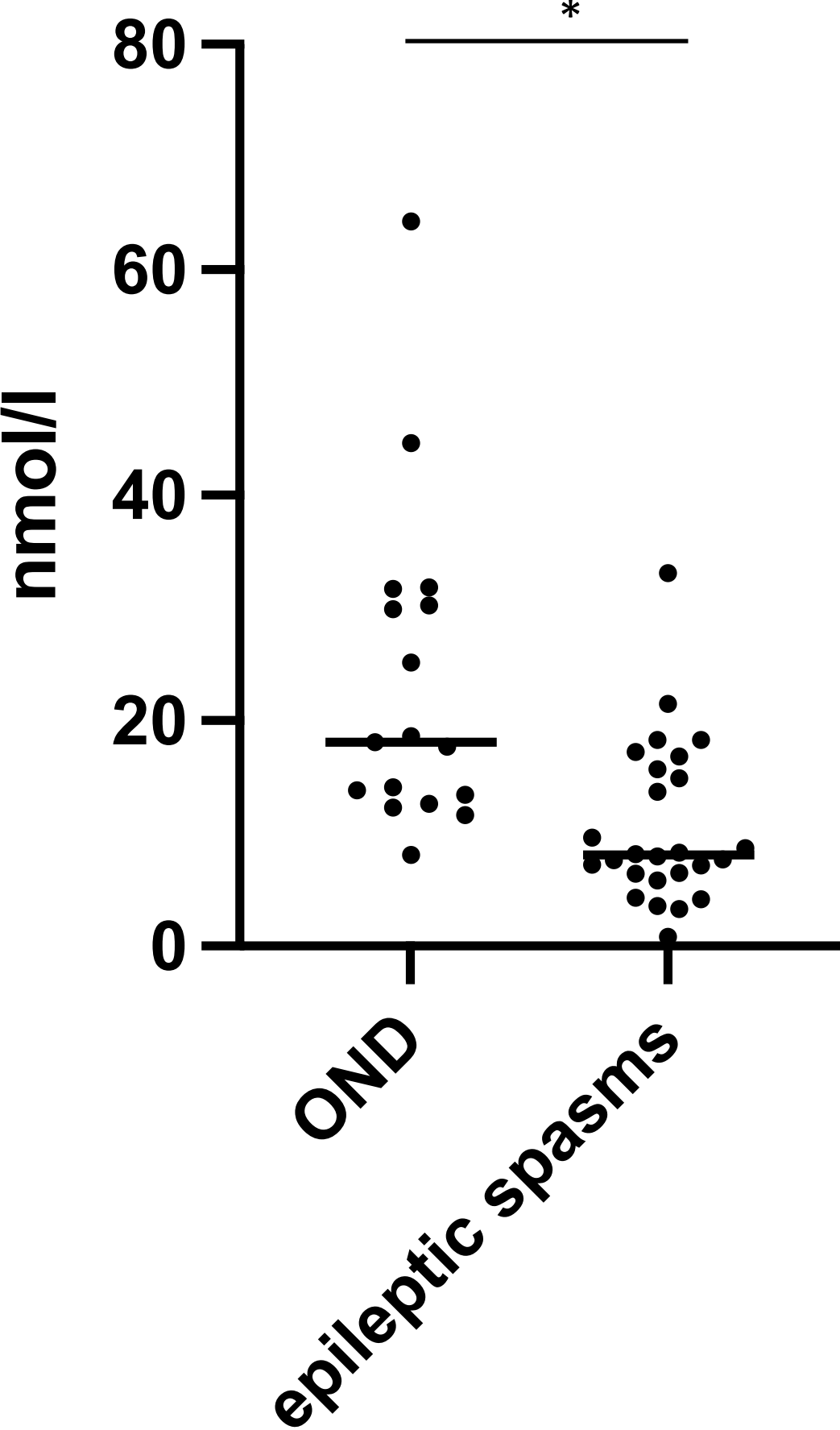

# KYNA/KYN <1yr

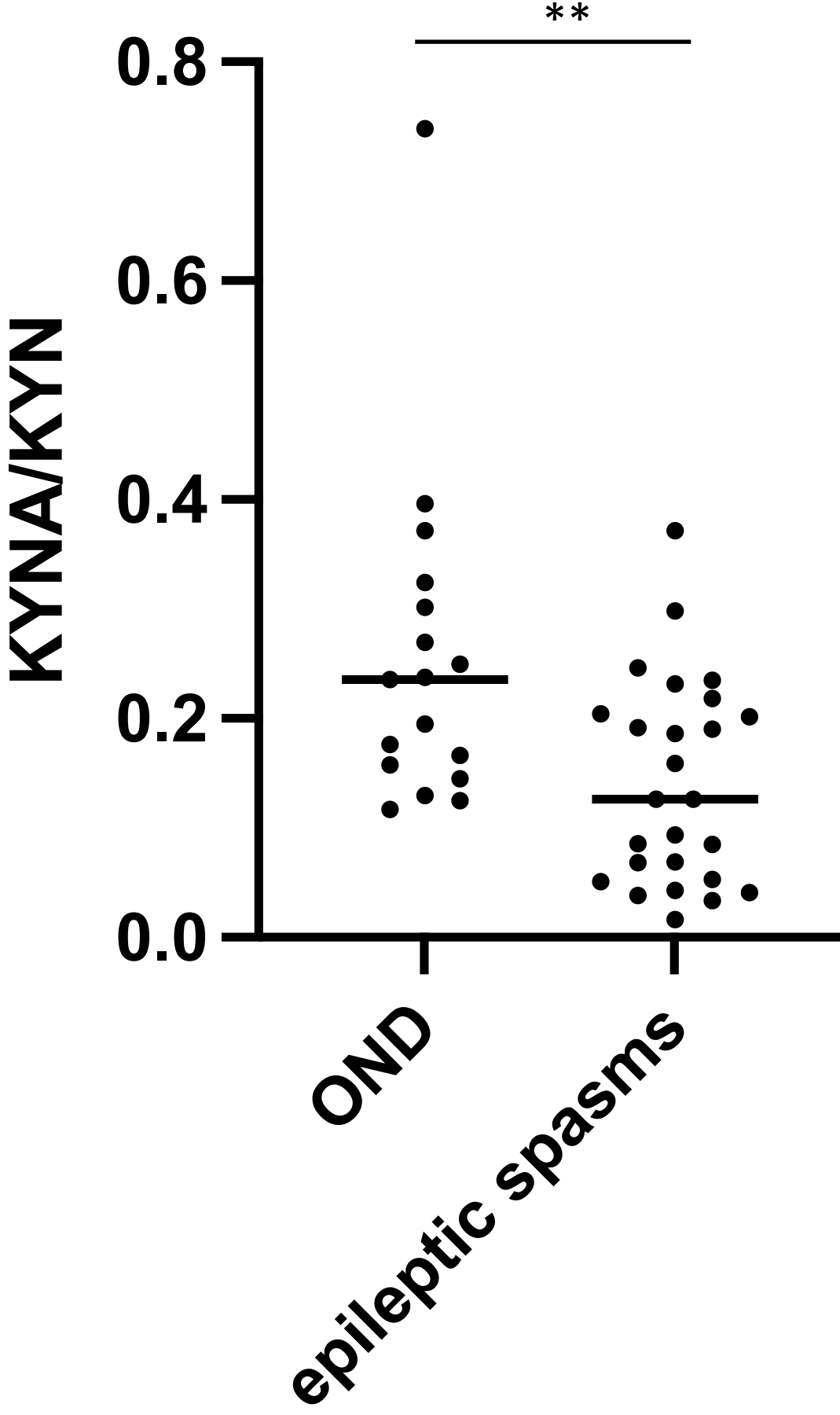

Supplement: Supplementary file 1 — Supplementary Figure 1. Analysis of patients under one year of age with OND (n=17, mean age 0.49 yr, median age 0.4 yr) and epileptic spasms (n=26, mean age 0.55yr, median age 0.5yr) showed age was not a factor driving the KYNA of KYNA/KYN changes in epileptic spasms. KYNA and KYNA/KYN ratio remained statistically lower in epileptic spasms group compared to OND group when only patients under 1 year were included (p=0.0003 (*) and p=0.0037 (**), respectively) (Mann Whitney U test). [file mmc1.pdf]

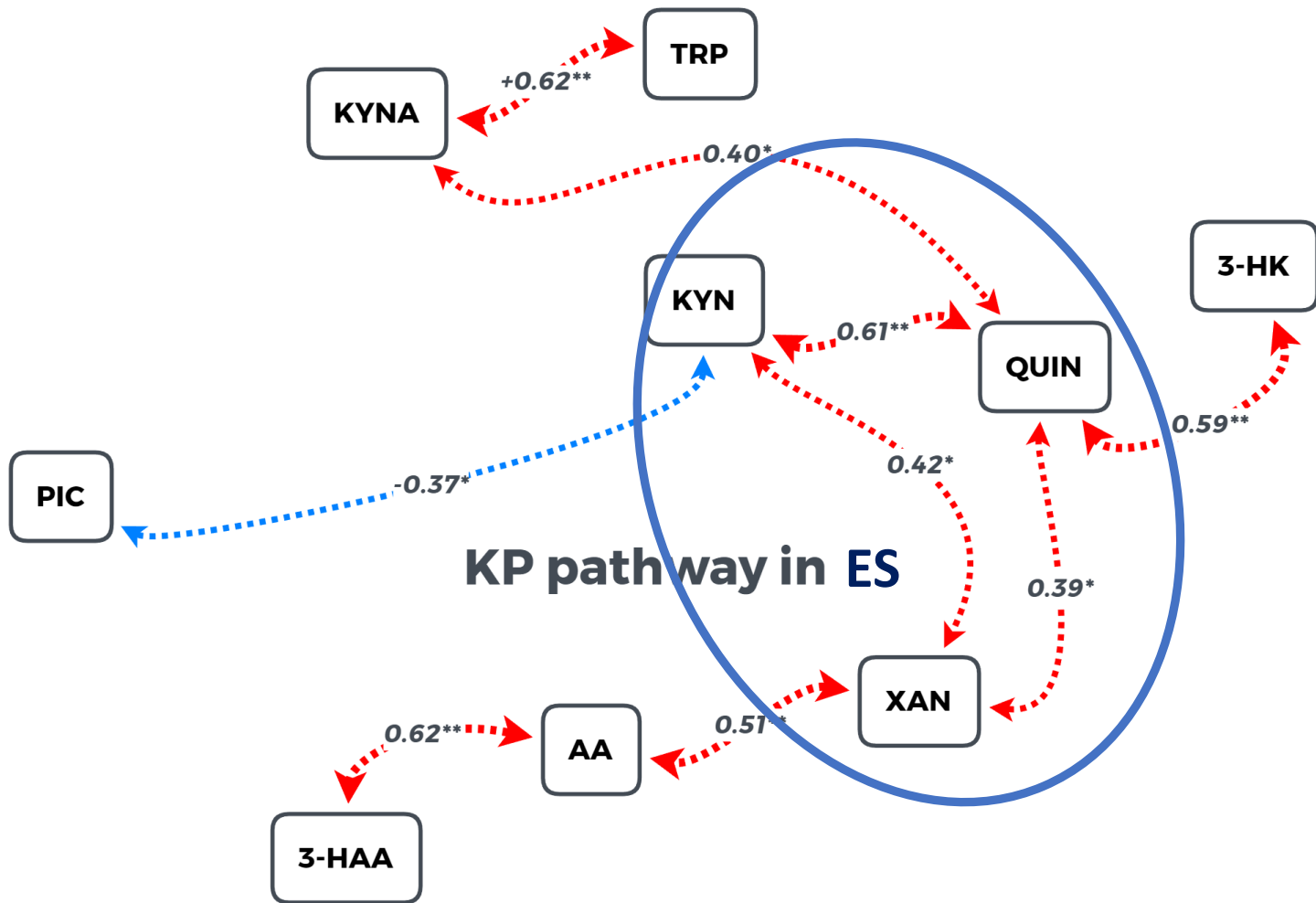

Supplement: Supplementary file 2 — Supplementary Figure 2: Examining the direction and the strength of the statistically significant correlations (Spearman's Rs) in the epileptic spasms group (ES), created in Xmind (https://www.xmind.net/download/). The stronger the correlation, the shorter the line is. Red lines show positive correlations, blue line shows negative correlation. Moderate strong positive correlations have Rs from 0.50 to 0.79, and weak positive correlations have Rs from 0.20 to 0.49. The most “connected” metabolite is QUIN which has multiple positive loops, with 2 stronger positive correlations (3-HK and KYN) and two weaker positive correlations (XAN and KYNA). KYNA has a moderate strong positive correlation with TRP. There are no correlations between TRP and KP pathway metabolites, other than KYNA. We hypothesise that this implies that by increasing the level of TRP (e.g., via diet), it is possible to increase the level of KYNA. There is weak negative correlation between KYN and PIC. [file mmc2.pdf]

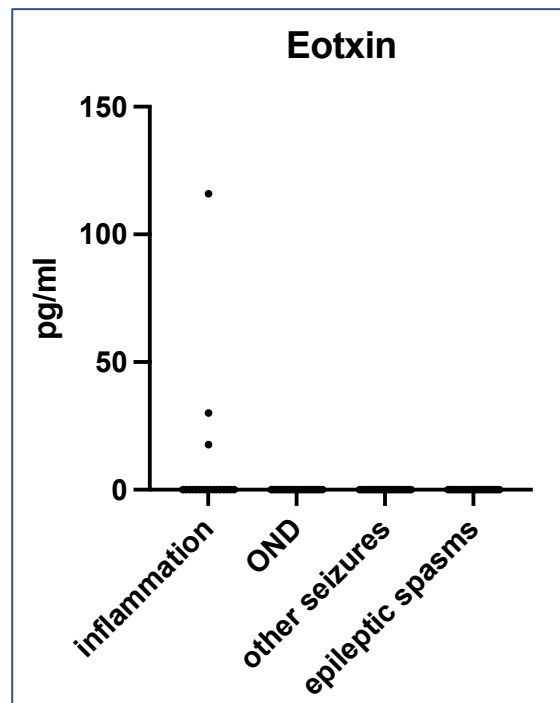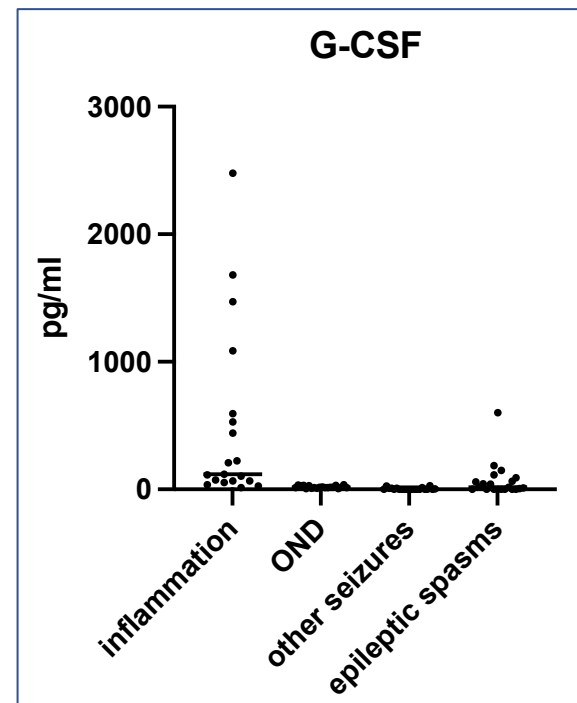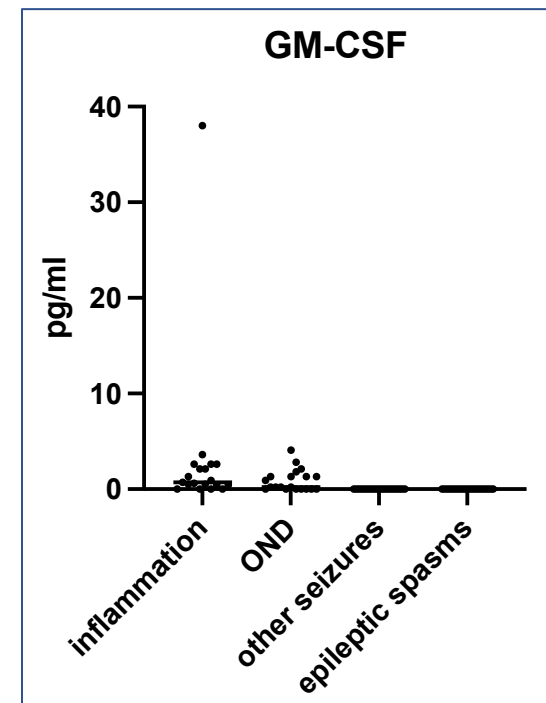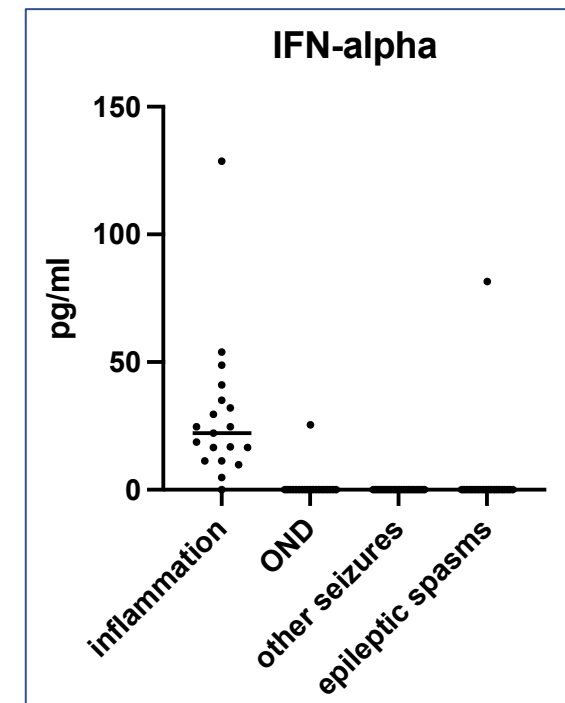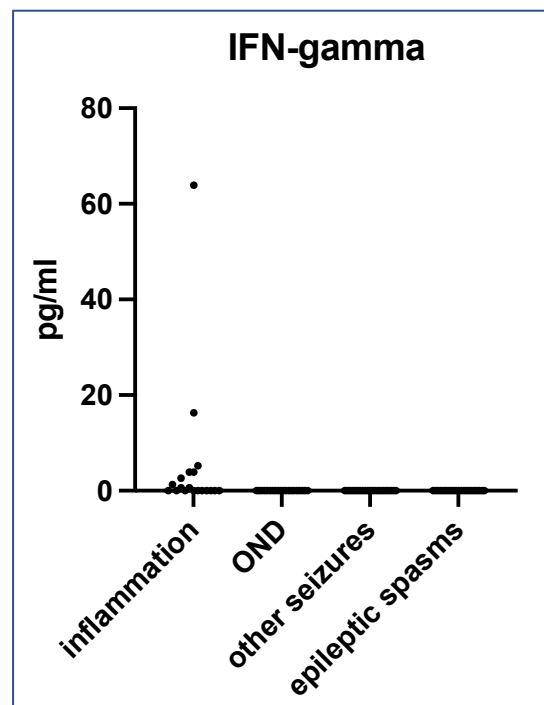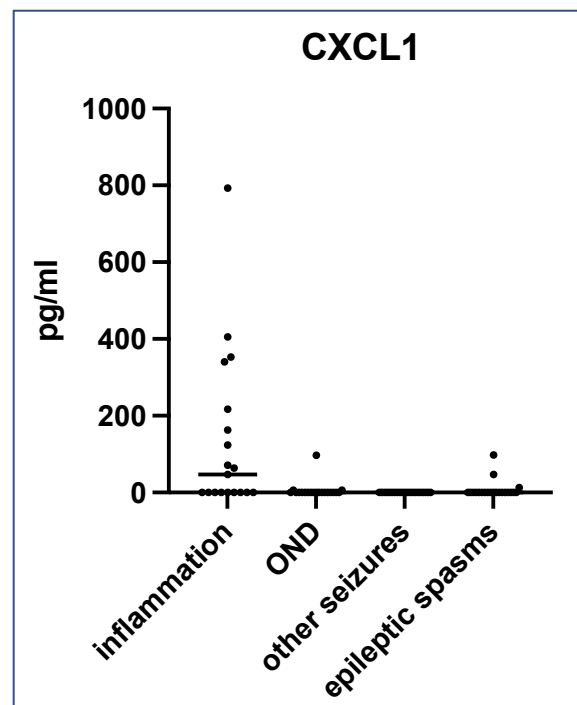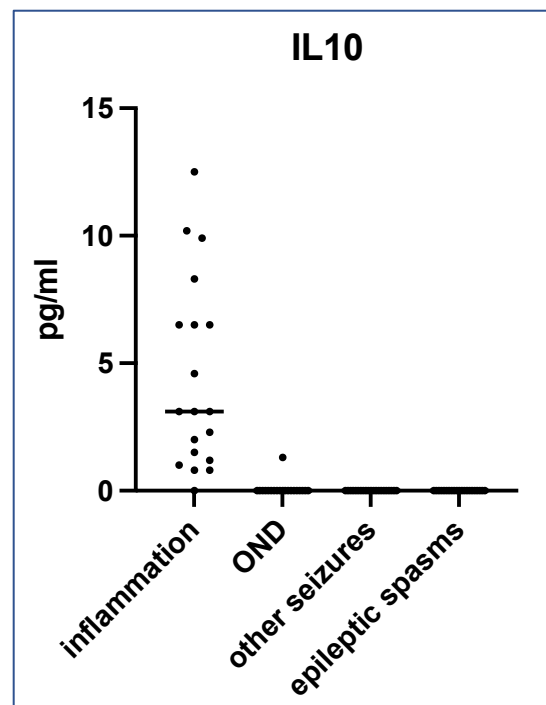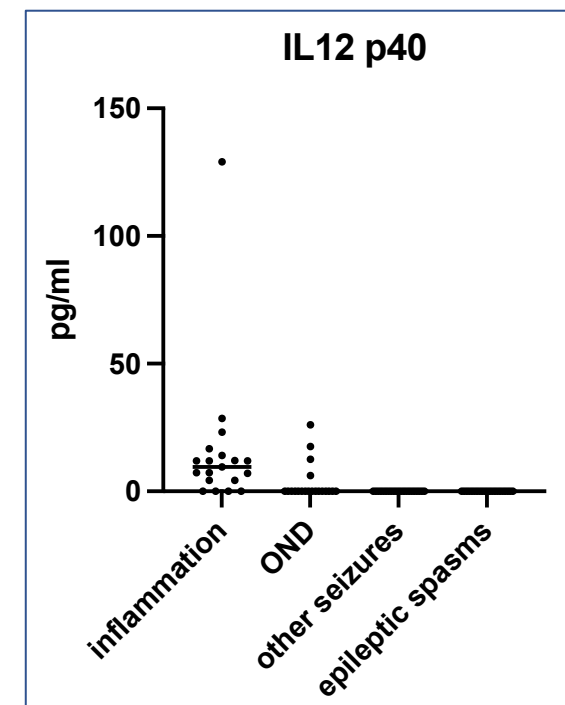

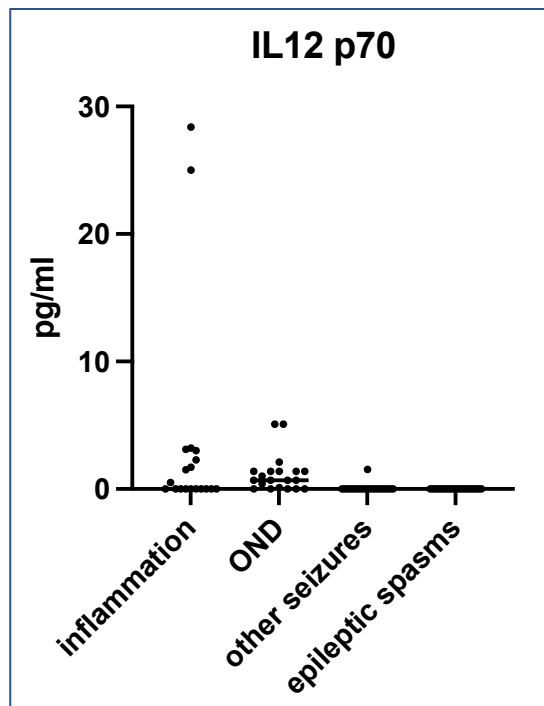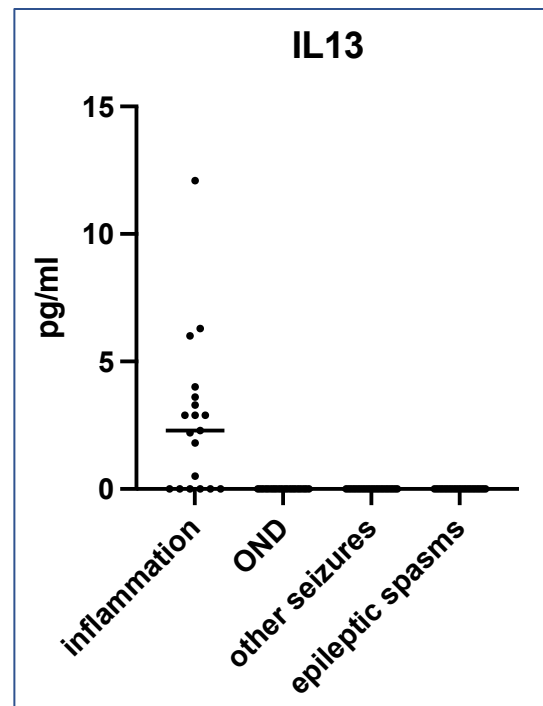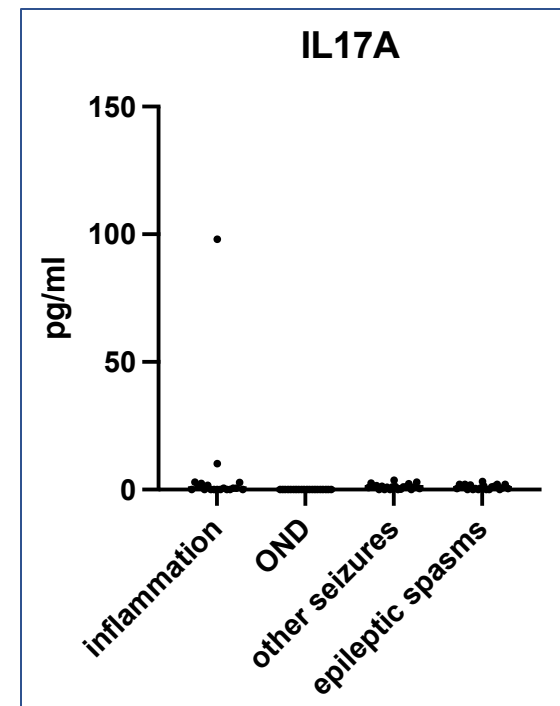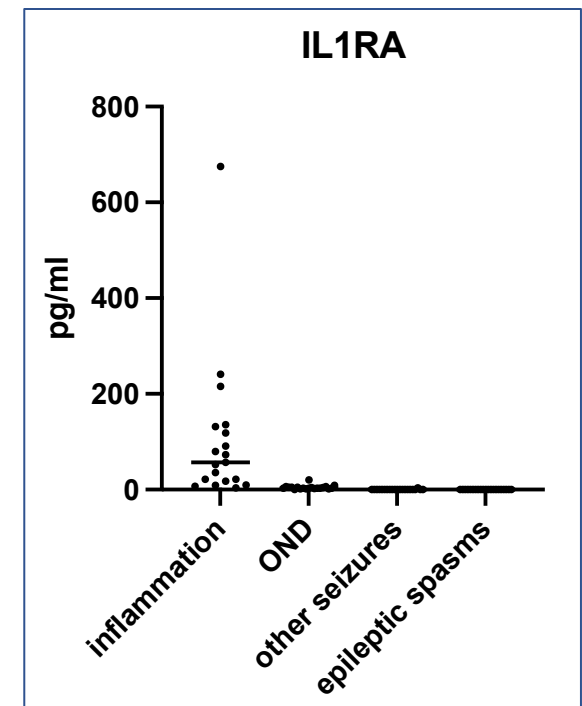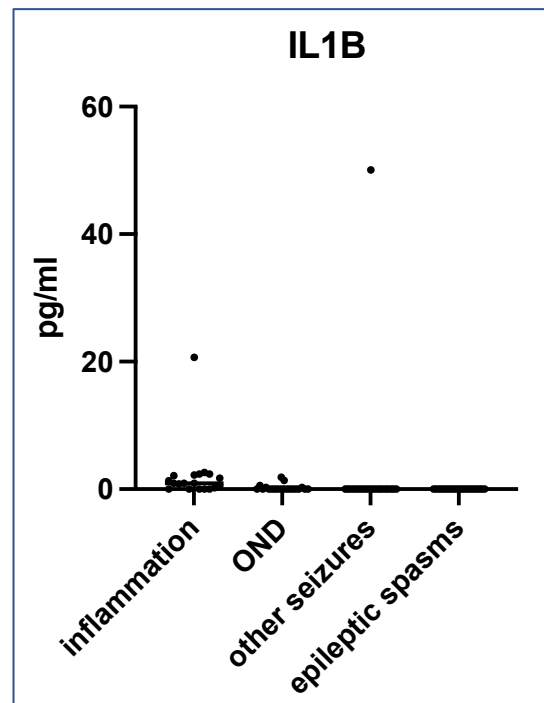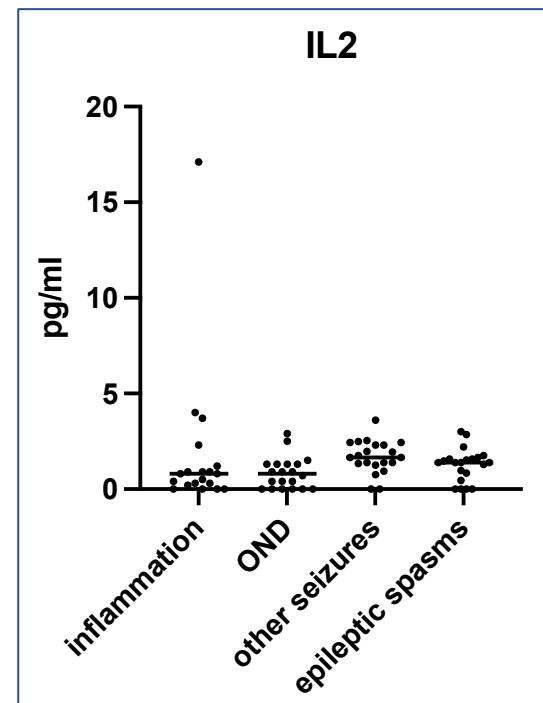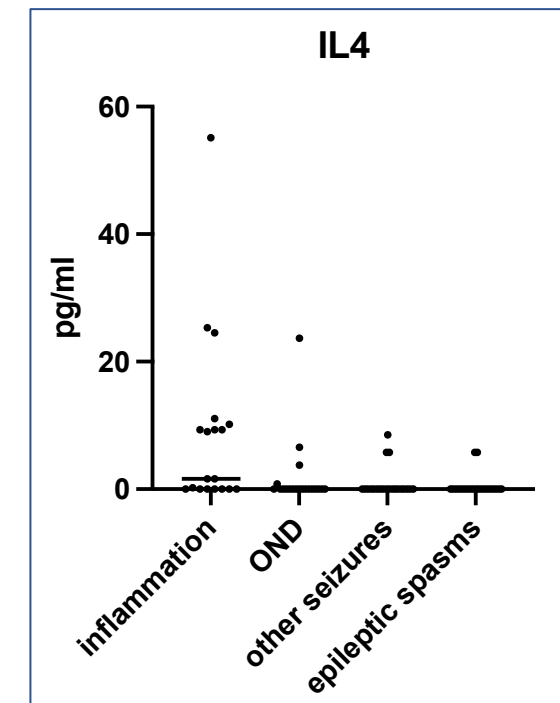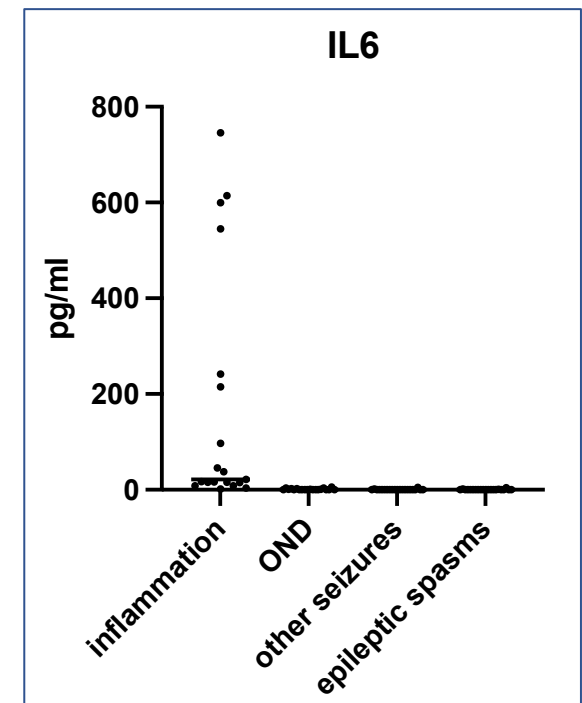

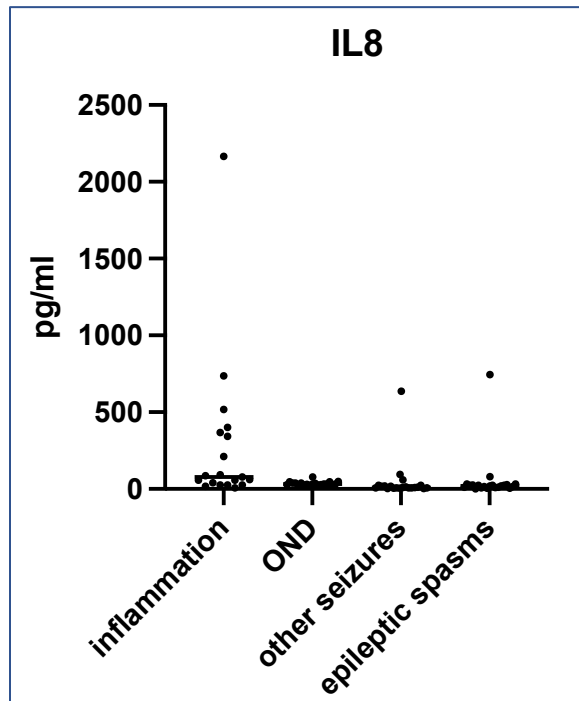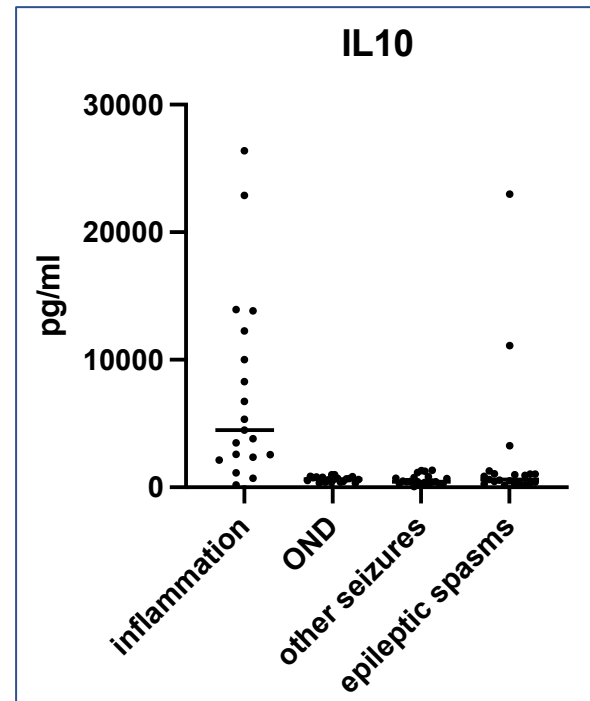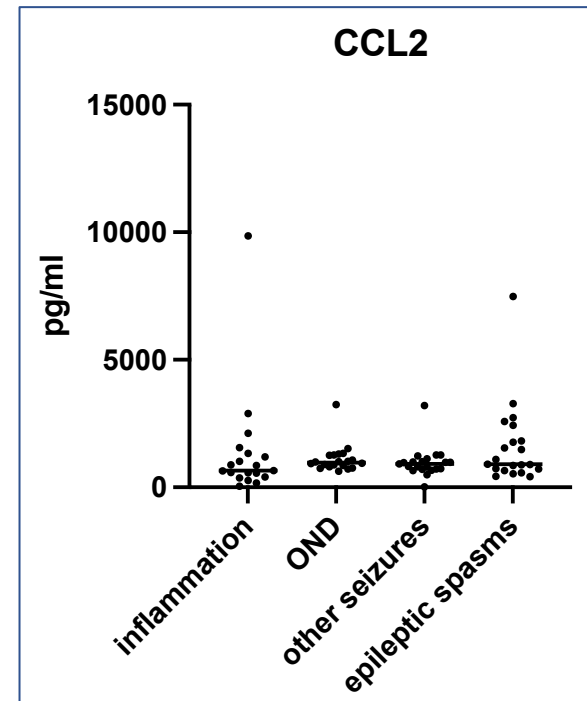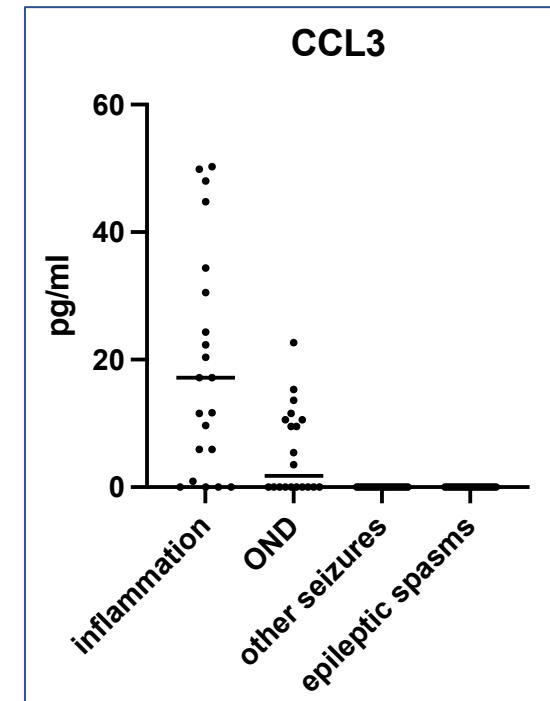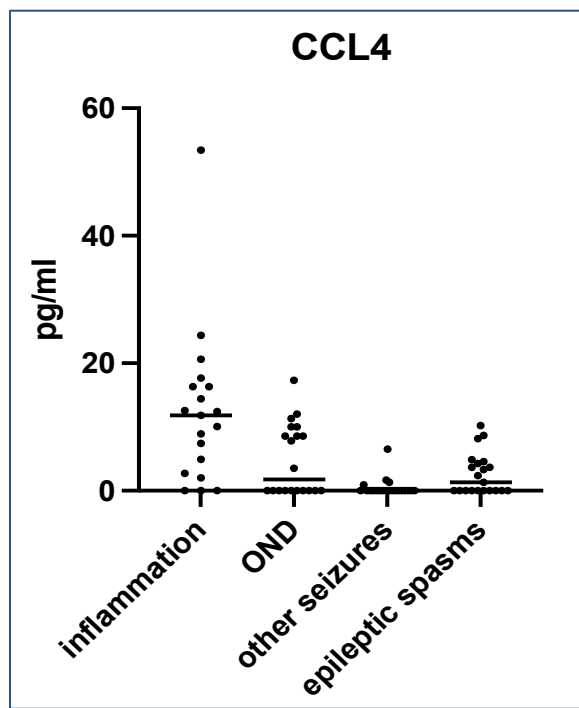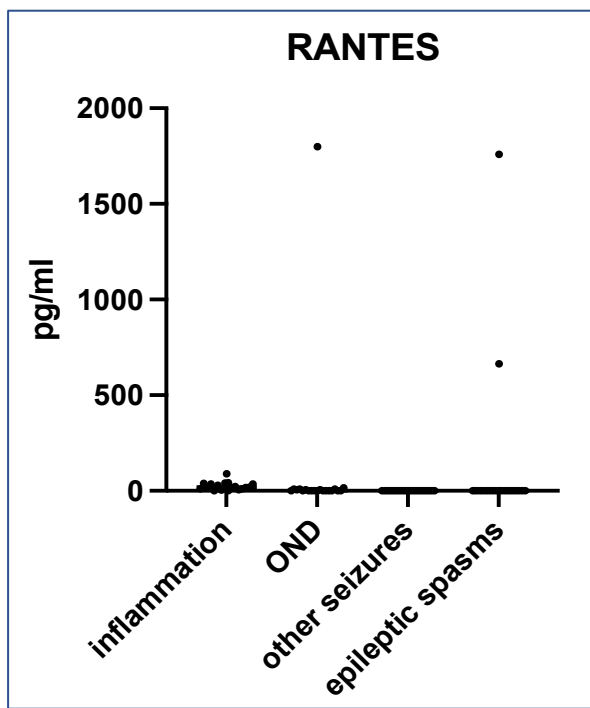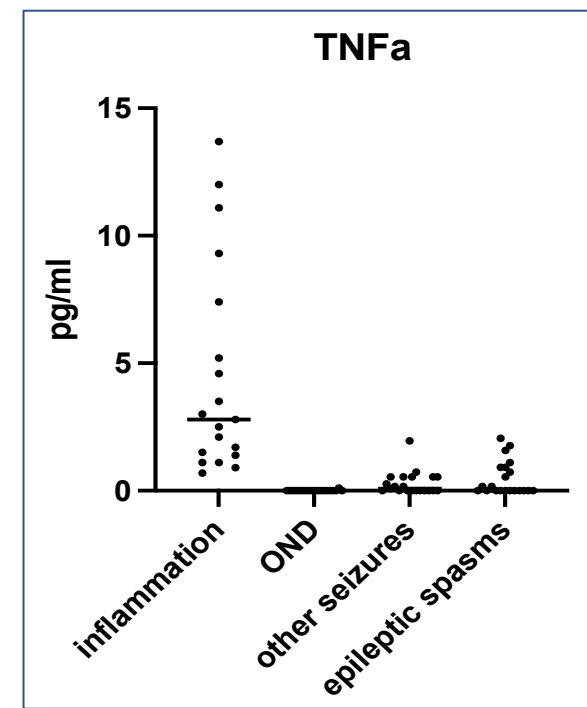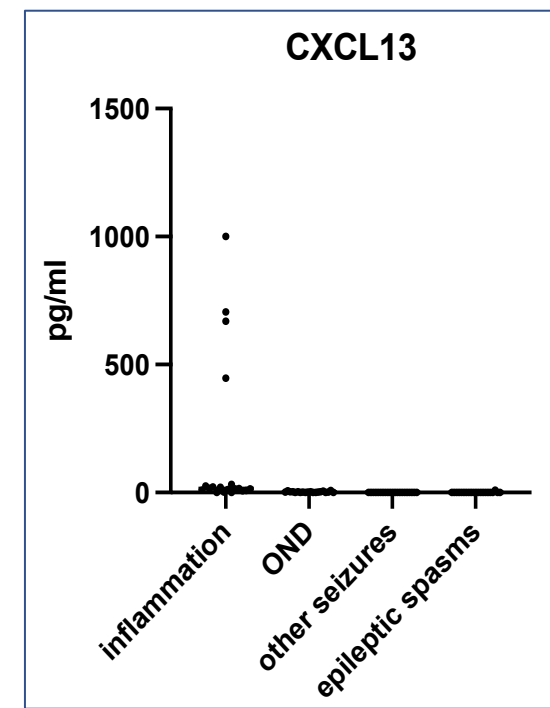

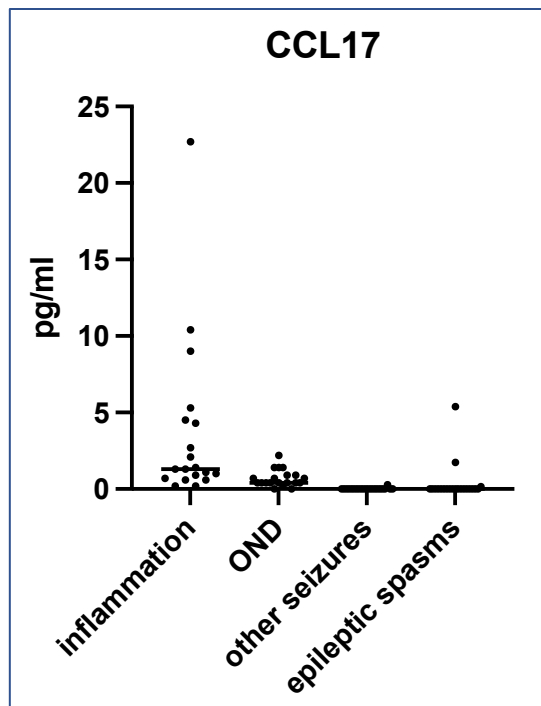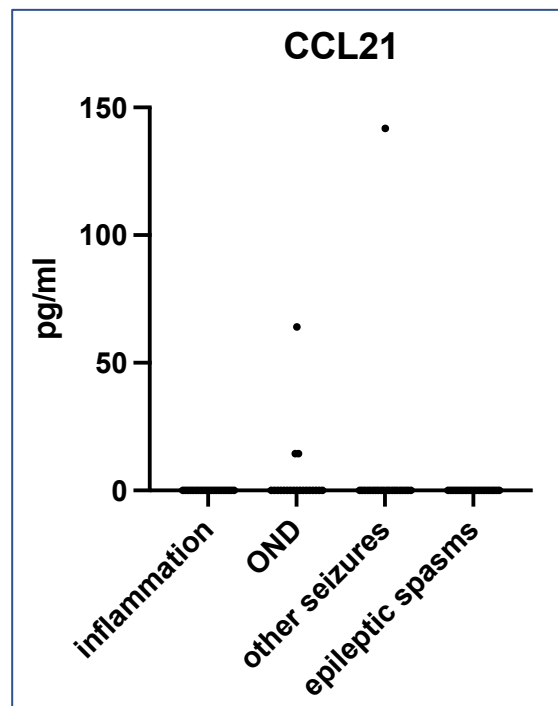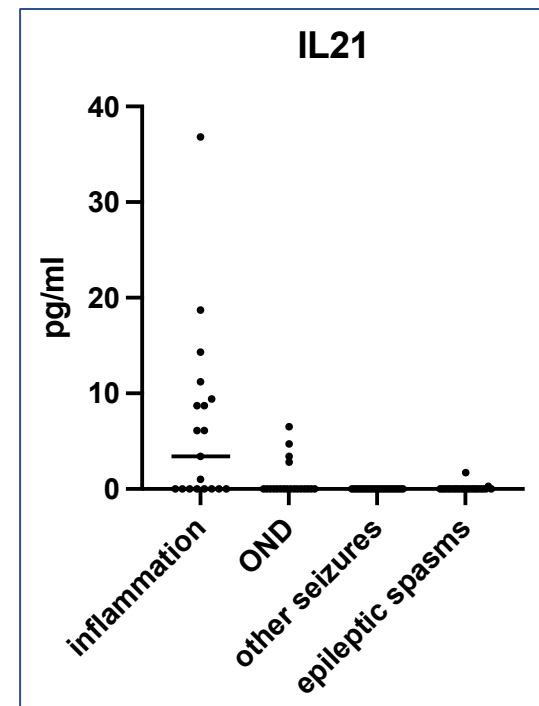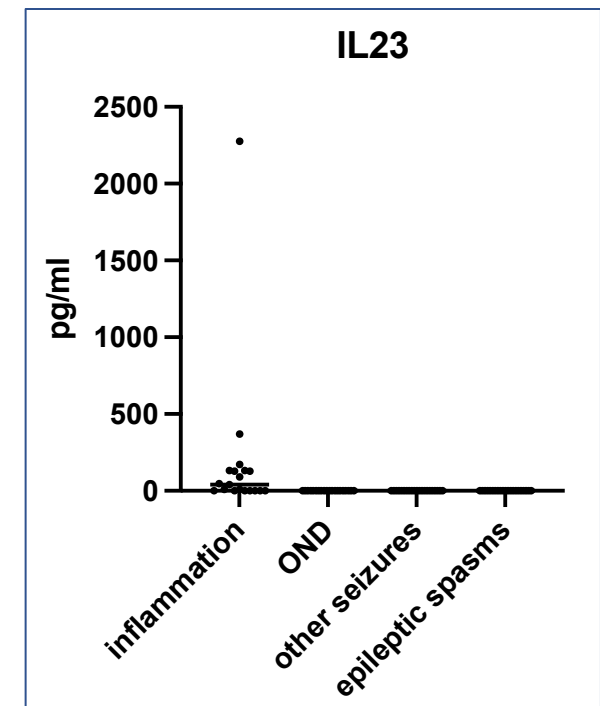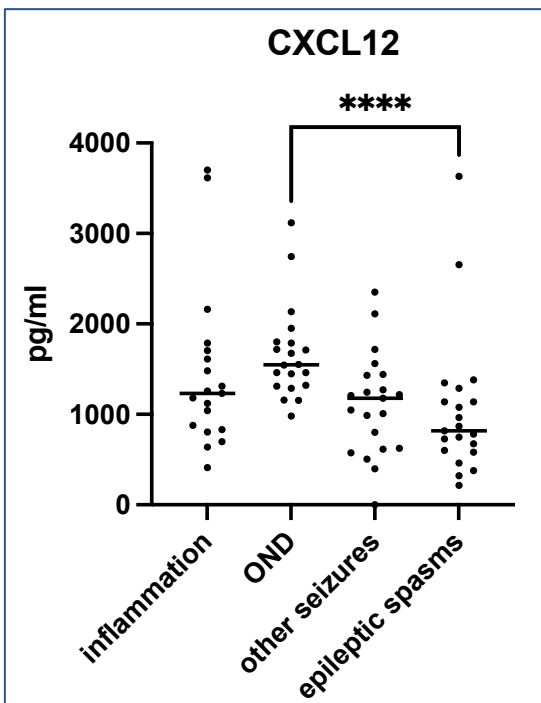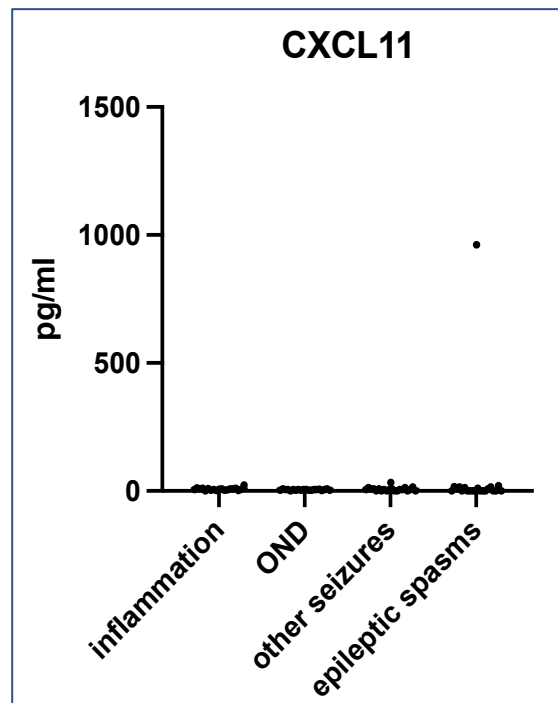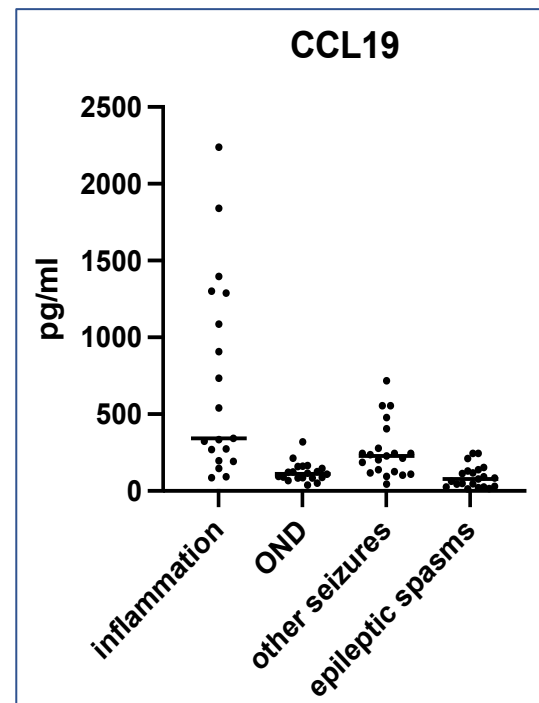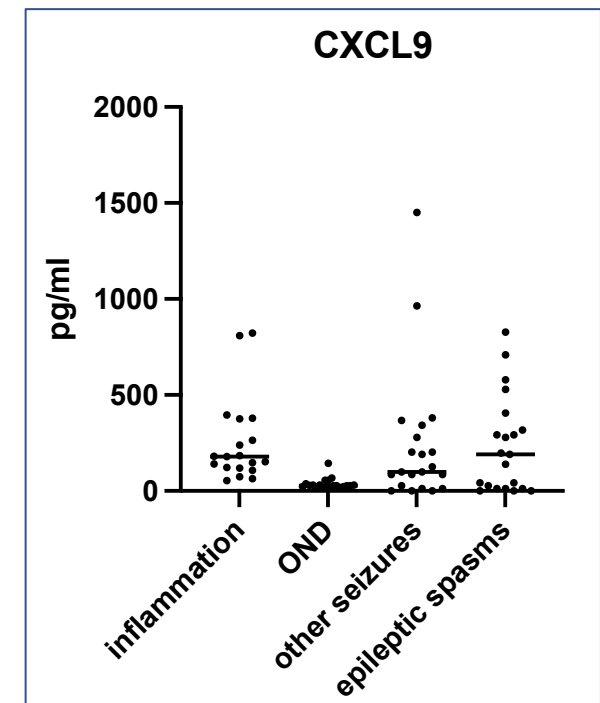

Supplement: Supplementary file 3 — Supplementary Figure 3. 32 cytokines and chemokines are presented in neuroinflammatory control group (n=19, 7 extra patients under 3 years old with encephalitis included for power), other non-inflammatory neurological controls (OND, n=20), other seizures (n=21) and epileptic spasms (n=21). The inflammatory neurological group had elevated cytokines/chemokines as expected. Cytokines and chemokines in epileptic spasms were compared with OND using pairwise Mann Whitney U test. Cytokines/chemokines were not statistically different in epileptic spasms compared to OND, apart from CXCL12 which was decreased in epileptic spasms compared to OND (p<0.0001) (Mann Whitney U test). [file mmc3.pdf]

**KYNA/KYN known aetiology**

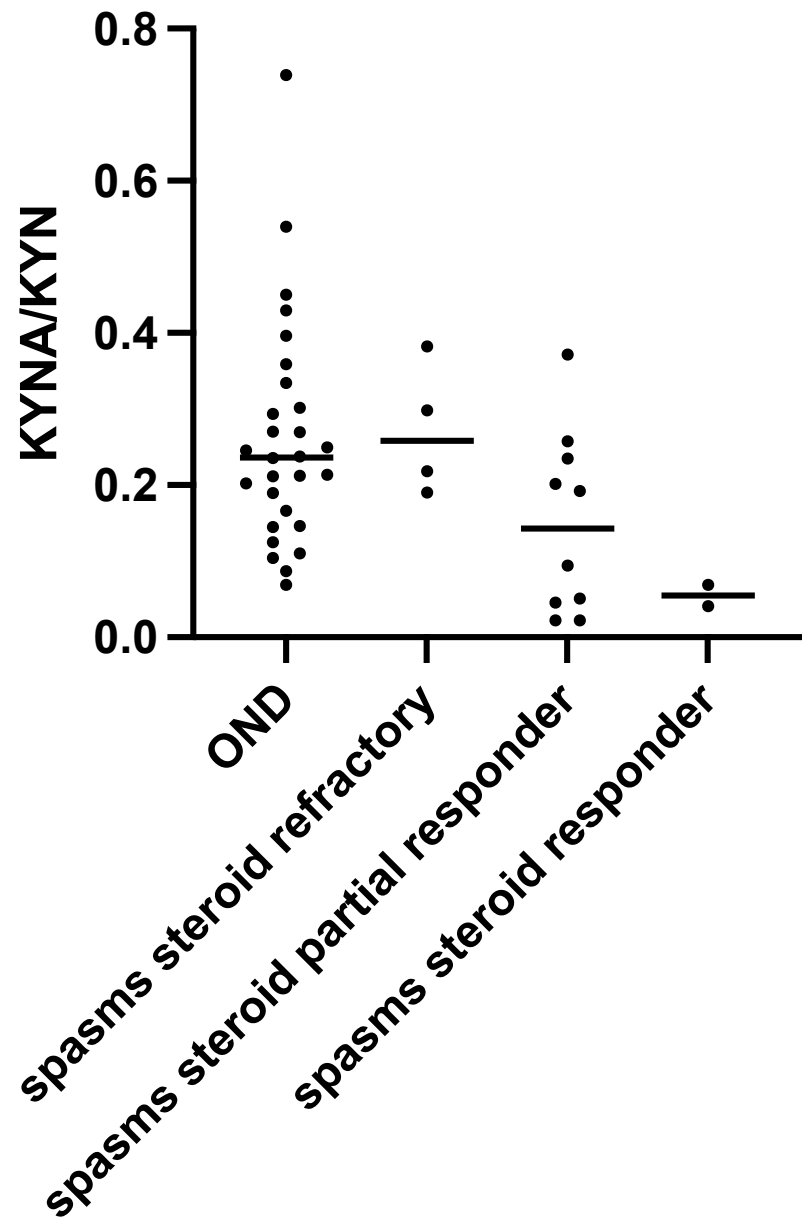

**KYNA/KYN unknown aetiology**

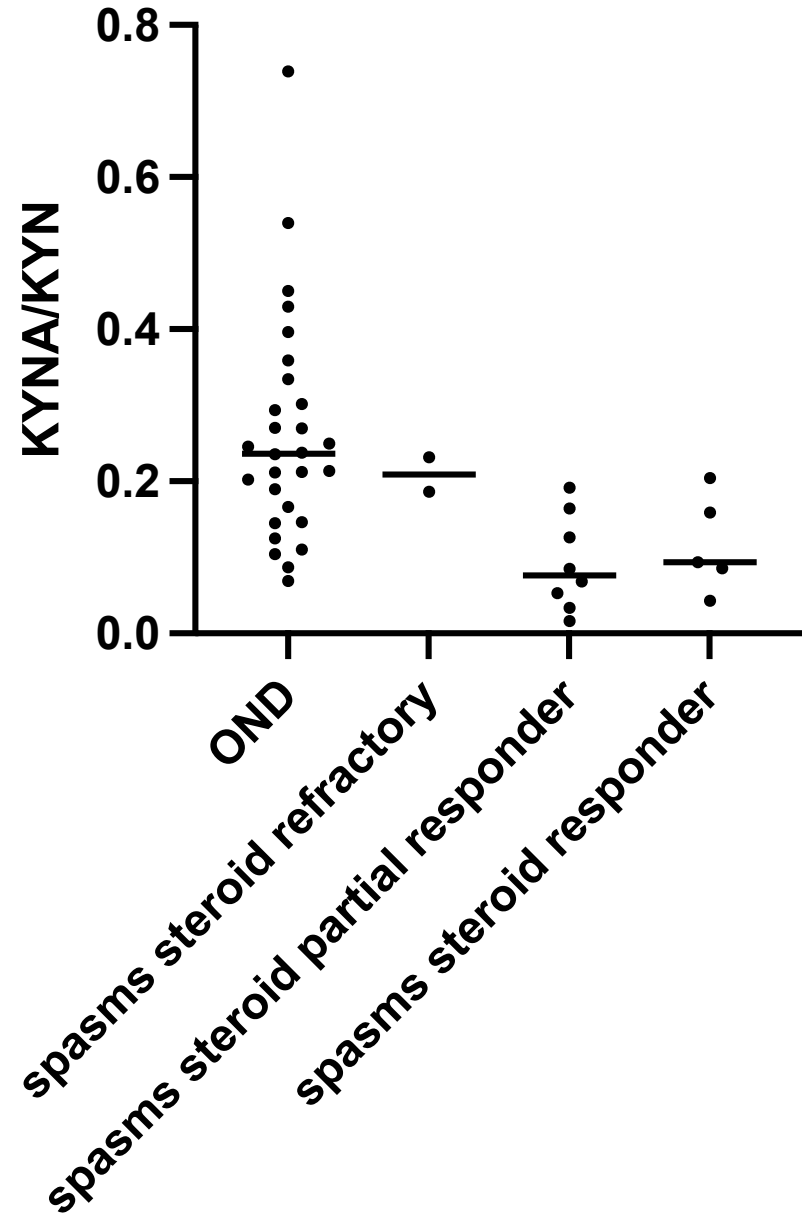

Supplement: Supplementary file 4 — Supplementary Figure 4. The analysis of epileptic spasms subgroups showed a clear trend of lower KYNA/KYN ratio associated with steroid responsiveness in both the known aetiology epileptic spasms group (gene-structural), and the unknown aetiology epileptic spasms group. [file mmc4.pdf]

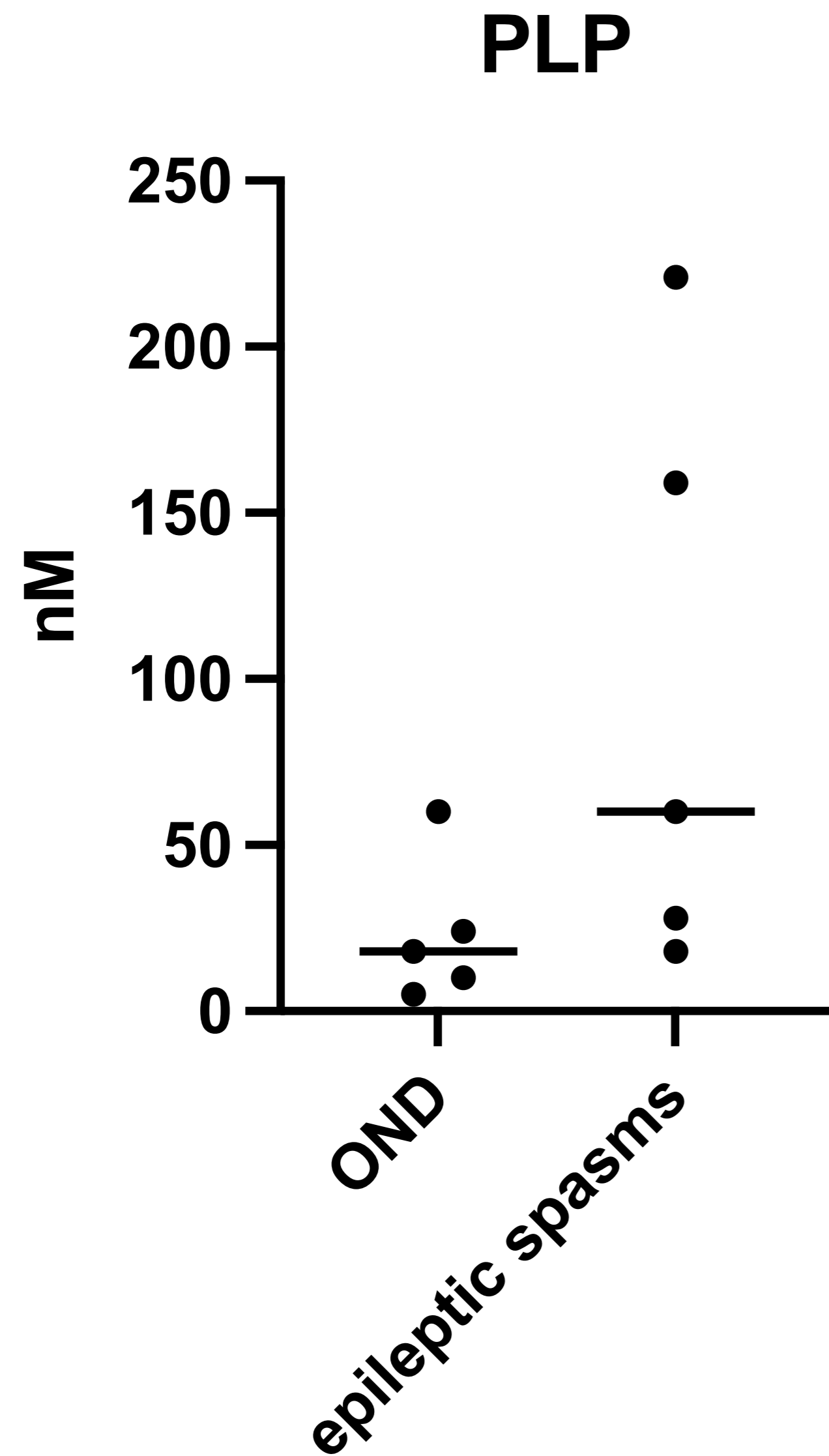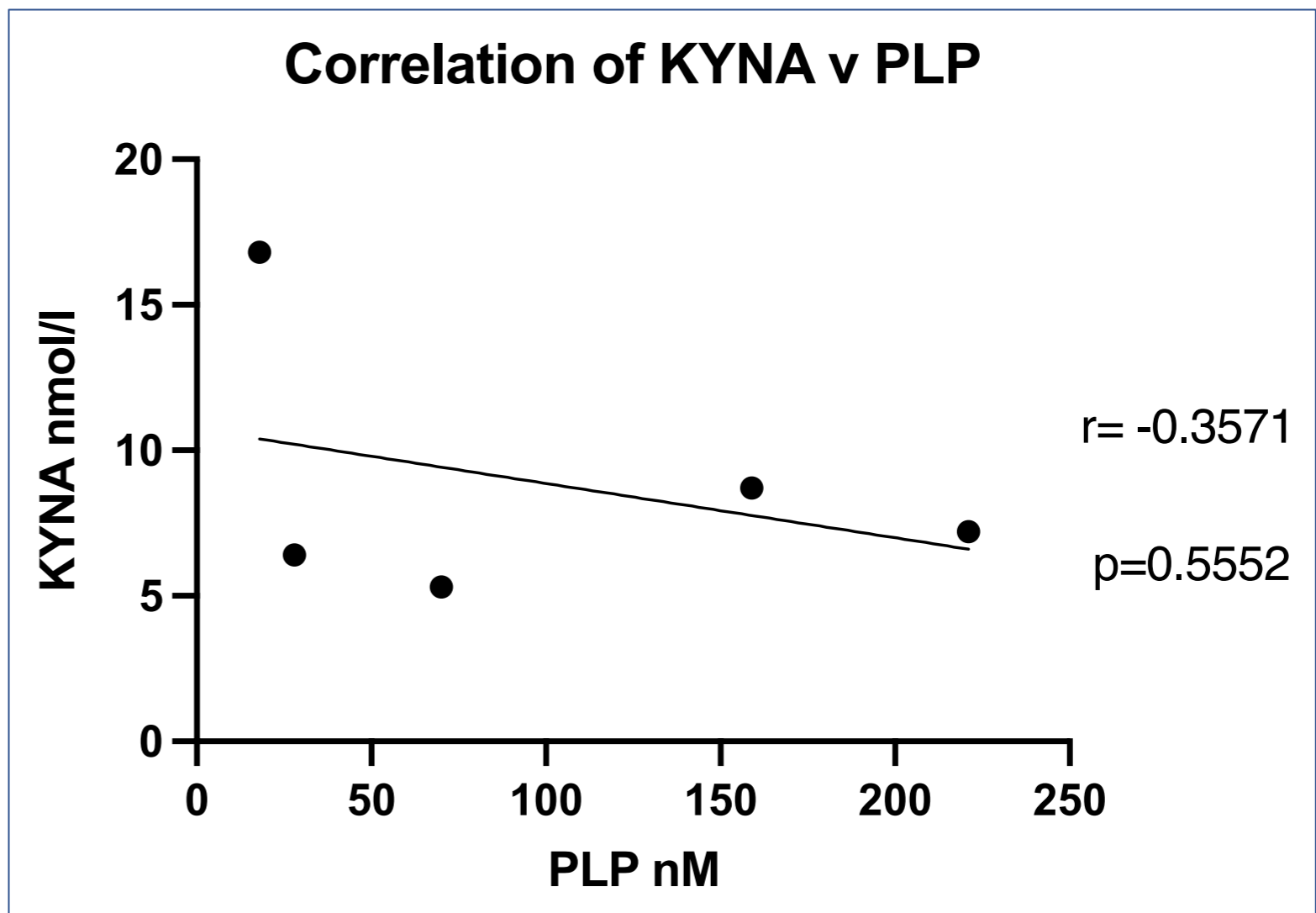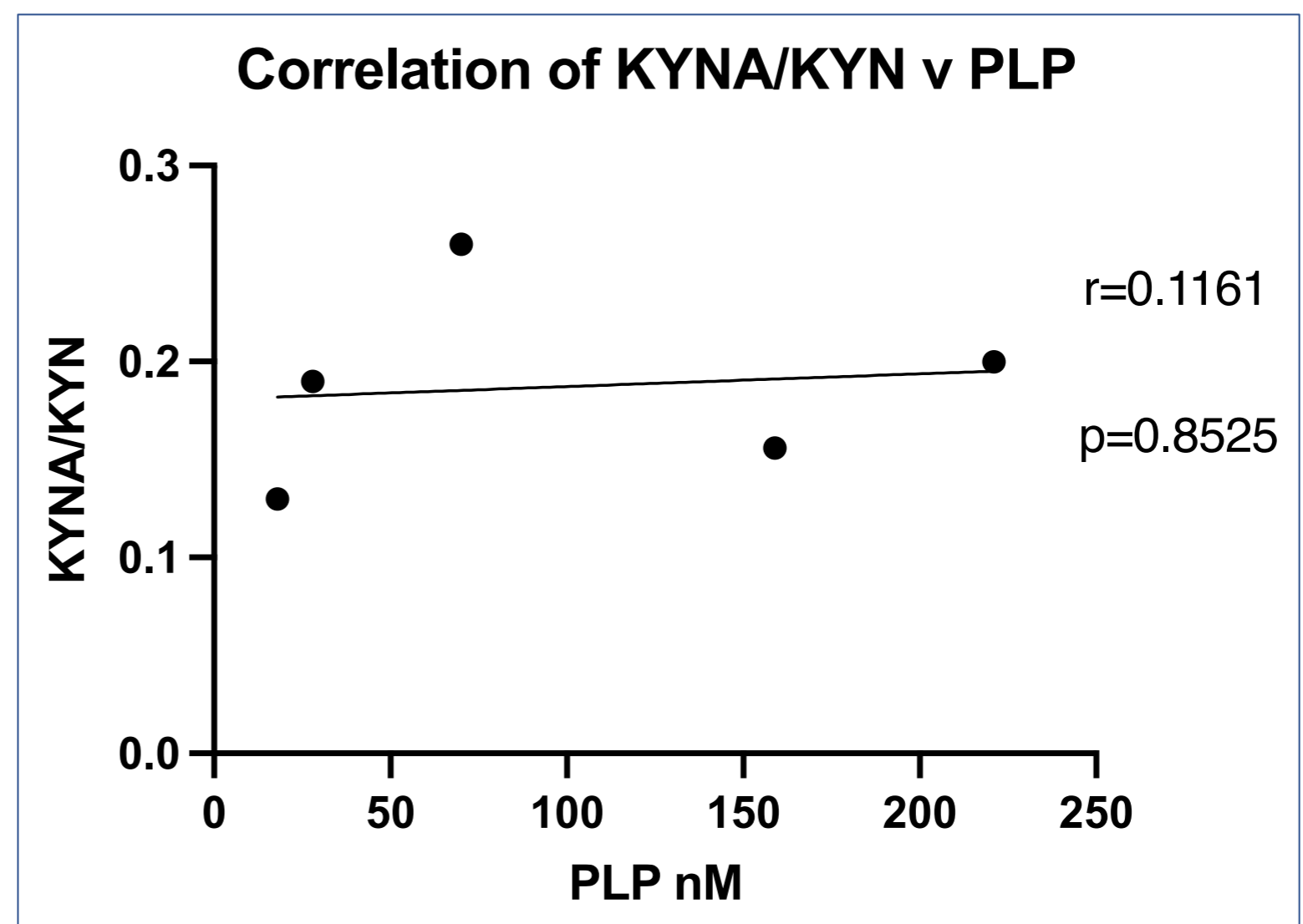

Supplement: Supplementary file 5 — Supplementary Figure 5. A comparison CSF pyridoxal 5’-phosphate (PLP) in epileptic spasms (n=5) and other non-inflammatory neurological controls (n=5) showed that PLP is not deficient in tested epileptic spasms patients, and the PLP concentration did not differ significantly in epileptic spasms compared to controls. Correlations of kynurenic acid and kynurenic acid/kynurenine ratio with pyridoxal 5’-phosphate revealed no statistical correlation. [file mmc5.pdf]
