# Supplementary material for: Decreased cerebrospinal fluid kynurenic acid in epileptic spasms: A biomarker of response to corticosteroids
Source: eBioMedicine. 2022 Sep 26;84:104280. doi: 10.1016/j.ebiom.2022.104280 (PMC9515432; doi:10.1016/j.ebiom.2022.104280)
Supplement: Supplementary file 6 [file mmc6.docx]

**Supplementary table 1.** Epileptic spasms (n=34) presented by aetiology subgroups (genetic, structural then unknown) with sex, age at spasms, duration of spasms at CSF, drugs at CSF, spasm aetiology, steroid response, age at follow-up and outcomes

| sex | Age spasm onset (yr) | Duration (yr) spasms at CSF | Drugs at CSF | Spasm aetiology group | Specific aetiology | Steroid response | Age last follow-up | Ongoing epilepsy | Developmental or intellectual outcome, motor outcome |
| --- | --- | --- | --- | --- | --- | --- | --- | --- | --- |
| F | 0.2 | 0.05 | - | Genetic | *CDKL5* | - | 2.4 | + | Severe ID, visual impair, CP |
| M | 0.5 | 0.3 | - | Genetic | *FOXG1* | Partial | 6 | - | ASD, mild ID |
| M | 0.9 | 0.1 | - | Genetic | Trisomy 21 | Partial | 3 | - | Moderate DD, ASD |
| M | 0.5 | 0.2 | - | Genetic | Leukoenceph. | None | 3 | + | Severe DD, dystonia |
| F | 1.0 | 0.5 | Lev, Clob, CBZ | Genetic | *TBCK* | Partial | 3.5 | + | Moderate DD |
| F | 1.3 | 0.1 | - | Genetic | *SCN2A* | Partial | 15 | + | Severe ID, CP |
| M | 1.3 | 0 | Clob | Genetic | *FOXG1* | Partial | 9 | + | Severe ID, CP |
| F | 1.4 | 0.2 | Lev | Genetic | 16p13.11 microdel | Partial | 3 | - | Moderate DD |
|  | | | | | | | | | |
| F | 0.36 | 0.3 | Nitraz | Structural | Occipital neonatal hypoglycaemic injury | Partial | 8.5 | + | Severe DD |
| M | 0.4 | 0.1 | - | Structural | Hemispheric malformation | none | 3.3 | + | Moderate DD, CP |
| F | 0.4 | 0.1 | - | Structural | Frontal FCD | yes | 6 | - | Moderate DD |
| M | 0.4 | 0.07 | - | Structural | Temporal type 1 FCD | yes | 9 | + | Moderate ID |
| M | 0.45 | 0.15 | - | Structural | Hypoxic ischaemic injury | Partial | 1.5 | - | Moderate DD |
| F | 0.5 | 0.2 | - | Structural | Hemispheric malformation | - | 6 | - | Mild language delay, CP |
| F | 0.6 | 0.1 | - | Structural | Temporal FCD | Partial | 0.8 | + | Normal |
| M | 0.6 | 0.1 | - | Structural | Temporal FCD | none | 4 | + | Speech delay |
| F | 1.1 | 0.2 | - | Structural | Temporal FCD | Partial | 2.5 | + | Moderate DD |
| M | 1.2 | 0.3 | - | Structural | Frontal FCD | none | 2.1 | + | Moderate DD |
| F | 2.85 | 0.15 | - | Structural | Previous HSV encephalitis | Partial | 4 | + | Mild ID, ASD |
|  | | | | | | | | | |
| M | 0.2 | 0.1 | - | Unknown | - | None | 5 | + | Moderate DD |
| M | 0.2 | 0.05 | - | Unknown | - | partial | 0.4 | Unk. | Unk. |
| M | 0.3 | 0.1 | - | Unknown | - | partial | 3 | - | Moderate DD |
| F | 0.34 | 0.1 | - | Unknown | - | partial | 3.5 | - | Mild speech delay |
| M | 0.35 | 0.1 | - | Unknown | - | partial | 4.5 | + | Moderate DD, ASD |
| F | 0.37 | 0.025 | - | Unknown | - | yes | 1.6 | - | Mild DD |
| M | 0.4 | 0.1 | - | Unknown | - | partial | 10 | - | Mild DD |
| M | 0.4 | 0.1 | - | Unknown | - | partial | 1.3 | + | Mild DD |
| M | 0.4 | 0.1 | - | Unknown | - | yes | 1 | - | Normal |
| M | 0.4 | 0.1 | - | Unknown | - | partial | 3.4 | - | Normal |
| F | 0.5 | 0.1 | VGB | Unknown | - | - | 3.7 | - | Mild DD |
| M | 0.55 | 0.05 | - | Unknown | - | yes | 1.8 | - | Mild speech delay |
| M | 0.6 | 0.1 | - | Unknown | - | None | 3 | + | Moderate DD |
| M | 0.65 | 0.05 | - | Unknown | - | yes | 6.5 | - | Moderate DD, ASD |
| F | 0.7 | 0.1 | - | Unknown | - | yes | 2 | - | Mild DD |

ASD: autistic spectrum disorder, Clob: clobazam, CP: cerebral palsy, CSF: cerebrospinal fluid, DD: developmental delay, FCD: focal cortical dysplasia, ID: intellectual disability, Leukoenceph: leukoencephalopathy, Lev: Leviteracetam, Nitraz: nitrazepam, Unk.: unknown, VGB: vigabatrin

**Supplementary Table 2.** Neuroinflammatory subgroup (n=12).

| Sex | Age at CSF (years) | OND subgroup | Specific aetiology |
| --- | --- | --- | --- |
| M | 0.8 | Inflammation | *SAMHD1* Aicardi Goutières syndrome |
| F | 0.8 | Inflammation | Pneumococcal meningitis |
| F | 1.2 | Inflammation | HHV6 encephalitis |
| M | 1.3 | Inflammation | Enteroviral encephalitis |
| M | 1.5 | Inflammation | Unknown encephalitis |
| F | 1.8 | Inflammation | Influenza encephalitis |
| F | 2 | Inflammation | Viral encephalitis in AML |
| F | 2.1 | Inflammation | Cerebellitis |
| F | 2.4 | Inflammation | Opsoclonus myoclonus ataxia syndrome |
| F | 2.5 | Inflammation | Enteroviral transverse myelitis |
| M | 2.5 | Inflammation | Acute encephalopathy with biphasic seizures (AESD) |

AML: acute myeloid leukaemia

**Supplementary Table 3.** Other non-inflammatory neurological (OND) controls (n=29). OND subgroups presented by age in subgroups: neurogenetic (n=15), symptomatic (n=7) and neurodevelopmental (n=7)

| Sex | Age at CSF (years) | OND subgroup | Specific aetiology |
| --- | --- | --- | --- |
| Female | 0.1 | Neurogenetic | Suspected genetic movement disorder |
| Male | 0.1 | Neurogenetic | Prader Willi syndrome |
| Male | 0.2 | Neurogenetic | Suspected genetic movement disorder |
| Male | 0.4 | Neurogenetic | *PNKP* gene mutation cerebral palsy, microcephaly, severe ID |
| Male | 0.7 | Neurogenetic | Congenital myasthenia |
| Male | 0.9 | Neurogenetic | Lissencephaly, ID, CP |
| Female | 0.9 | Neurogenetic | Sepiapterin reductase deficiency |
| Female | 1 | Neurogenetic | *MAP2K2* cardiocraniofacial syndrome |
| Male | 1.6 | Neurogenetic | Dev Delay, bilateral blindness, cerebral atrophy |
| Female | 1.6 | Neurogenetic | Cockayne syndrome |
| Male | 1.7 | Neurogenetic | Riboflavin transporter deficiency (SLC52A2 mutation) |
| Male | 2.7 | Neurogenetic | ASD, receptive language delay, microdeletion |
| Female | 2.8 | Neurogenetic | Suspected stiff gait, blepharospasm |
| Female | 2.9 | Neurogenetic | Dopa responsive dystonia |
| Male | 3 | Neurogenetic | Christinsen syndrome |
|  | | | |
| Female | 0.6 | Symptomatic (structural) | Cerebral palsy |
| Female | 0.8 | Symptomatic (structural) | Dysplastic cerebellar gangliocytoma (Lhermitte-Duclos) |
| Male | 1.6 | Symptomatic (structural) | Cerebral palsy due to placental abruption |
| Male | 1.6 | Symptomatic (structural) | Cerebral palsy |
| Male | 1.6 | Symptomatic (structural) | Raised intracranial pressure- shunt insertion |
| Male | 2 | Symptomatic (structural) | Traumatic head injuries with post concussive language change |
| Female | 2.4 | Symptomatic (structural) | Suspected dystonic CP |
|  |  |  |  |
| Male | 0.3 | Neurodevelopment | Paroxysmal episodes eye movements |
| Male | 0.5 | Neurodevelopment | Ocular flutter and delayed development |
| Female | 1.4 | Neurodevelopment | Austistic regression |
| Female | 1.5 | Neurodevelopment | ASD regression 1.5 |
| Male | 2.2 | Neurodevelopment | ASD regression at 2 years |
| Male | 2.5 | Neurodevelopment | Moderate developmental delay |
| Male | 2.9 | Neurodevelopment | Paroxysmal stereotypical movements |

ASD: autistic spectrum disorder, CP: cerebral palsy, ID: intellectual disability

**Supplementary Table 4.** Other seizures and epilepsy (not epileptic spasms) group by subgroups, status epilepticus (n=7), developmental epileptic encephalopathy (n=11), suspected genetic epilepsy not otherwise specified (nos) (n=5), symptomatic epilepsy (n=3)

| Sex | Age at CSF (years) | OND subgroup | Specific aetiology |
| --- | --- | --- | --- |
| F | 0.6 | Status epilepticus | Afebrile status epilepticus |
| F | 0.67 | Status epilepticus | Febrile status epilepticus |
| F | 1 | Status epilepticus | Febrile status epilepticus |
| M | 1.1 | Status epilepticus | Febrile status epilepticus |
| F | 1.75 | Status epilepticus | Febrile status epilepticus |
| M | 1.8 | Status epilepticus | Febrile status epilepticus |
| M | 2.2 | Status epilepticus | Afebrile status epilepticus |
|  | | | |
| F | 0.3 | Genetic DEE | SCN2A DEE |
| F | 0.4 | Genetic DEE | GLUT1 DEE |
| F | 0.5 | Genetic DEE | STXBP1 DEE |
| F | 0.7 | Genetic DEE | SCN2A DEE |
| M | 0.7 | Genetic DEE | SCN8A DEE |
| F | 0.83 | Genetic DEE | SCN8A DEE |
| M | 0.9 | Genetic DEE | GRIN1 DEE |
| F | 1.5 | Genetic DEE | FOXG1 DEE |
| F | 2 | Genetic DEE | SCN8A DEE |
| F | 2.9 | Genetic DEE | DEE, no gene identified |
| F | 2.9 | Genetic DEE | Angelman syndrome, epilepsy |
|  | | | |
| F | 1.4 | Genetic epilepsy nos | Suspected genetic epilepsy |
| F | 1.5 | Genetic epilepsy nos | Suspected genetic epilepsy |
| M | 1.7 | Genetic epilepsy nos | Focal epilepsy |
| M | 2.7 | Genetic epilepsy nos | Drop attacks, epilepsy, suspected genetic |
| M | 3 | Genetic epilepsy nos | Myoclonic astatic epilepsy |
|  | | | |
| F | 0.8 | Symptomatic | HIE associated refractory epilepsy |
| F | 1.5 | Symptomatic | Focal epilepsy due to FCD |
| M | 2.1 | Symptomatic | Focal epilepsy due to FCD |

CSF: cerebrospinal fluid, DEE: developmental epileptic encephalopathy, FCD: focal cortical dysplasia, HIE: hypoxic ischaemic encephalopathy, nos: not otherwise specified.

**Supplementary Table 5.** Percentage of IS patients and control samples above the lower detection limit.

| **Analyte** | **Infantile Spasms (%)** | **Other epilepsy (%)** | **Neurological Inflammatory (%)** | **Non-inflammatory neurological group (%)** |
| --- | --- | --- | --- | --- |
| Neopterin | 100 | 100 | 100 | 100 |
| Tryptophan | 100 | 100 | 100 | 96 |
| Kynurenine | 100 | 100 | 100 | 96 |
| Kynurenic acid | 100 | 100 | 100 | 96 |
| 3-hydroxykynurenine | 74 | 85 | 67 | 76 |
| Xanthurenic acid | 97 | 96 | 92 | 90 |
| Anthranilic acid | 82 | 77 | 92 | 80 |
| 3-hydroxyanthranilic acid | 60 | 65 | 92 | 83 |
| Quinolinic acid | 100 | 100 | 100 | 96 |
| Picolinic acid | 97 | 88 | 77 | 90 |

**Supplementary Table 6:** Table of pairwise statistical comparisons of metabolites using non-parametric Mann Whitney U test: Epileptic spasms v control groups. Raw p values are presented. There are 3 pairwise comparisons for all 9 metabolites. Including Bonferroni correction, p values <0.0018 are highlighted in bold and presented in Figure 2.

| Metabolite | Epileptic spasms v inflammation | Epileptic spasms v OND | Epileptic spasms v other seizures |
| --- | --- | --- | --- |
| Kynurenic acid | **<0.0001** | **0.0001** | 0.0077 |
| Tryptophan | 0.3661 | 0.6889 | 0.3386 |
| Kynurenine | **<0.0001** | 0.7440 | 0.8532 |
| 3-hydroxykynurenine | 0.0175 | 0.8315 | 0.1640 |
| Xanthurenic acid | 0.0170 | 0.1418 | 0.0122 |
| Anthranilic acid | 0.8358 | 0.0243 | 0.4334 |
| 3-hydroxyanthanthranilic acid | 0.8792 | 0.5554 | 0.4022 |
| Quinolinic acid | **<0.0001** | 0.7547 | 0.7813 |
| Picolinic acid | 0.9816 | 0.6630 | 0.3104 |

**Supplementary Table 7:** Table of pairwise statistical comparisons of ratios using non-parametric Mann Whitney U test: Epileptic spasms v control groups. Raw p values are presented. There are 3 pairwise comparisons for all 7 ratios. Including Bonferroni correction, p values <0.0024 are highlighted in bold and presented in Figure 3.

| Ratio | Epileptic spasms v inflammation | Epileptic spasms v OND | Epileptic spasms v other seizures |
| --- | --- | --- | --- |
| KYNA/KYN | 0.8532 | **0.0002** | 0.1510 |
| KYN/TRP | **0.0001** | 0.5861 | 0.5609 |
| 3HK/KYN | 0.99 | 0.5638 | 0.1707 |
| AA/KYN | 0.0036 | 0.0867 | 0.1791 |
| XAN/3HK | 0.0040 | 0.2300 | 0.0033 |
| 3HAA/3HK | 0.1969 | 0.8077 | 0.8388 |
| 3HAA/AA | 0.8042 | 0.3697 | 0.1743 |

**Supplementary Table 8:** Table of pairwise statistical comparisons of values using non-parametric Mann Whitney U test. Steroid responsiveness by group is compared. Raw p values are presented.

| Metabolite or ratio | steroid responder v steroid refractory | Steroid partial responder v steroid refractory |
| --- | --- | --- |
| KYNA | 0.187 | 0.0183 |
| KYNA/KYN | 0.0047 | 0.0183 |
